# Genome-wide survey of prokaryotic serine proteases: Analysis of distribution and domain architectures of five serine protease families in prokaryotes

**DOI:** 10.1186/1471-2164-9-549

**Published:** 2008-11-19

**Authors:** Lokesh P Tripathi, R Sowdhamini

**Affiliations:** 1 National Centre for Biological Sciences, TIFR, GKVK Campus, Bellary Road, Bangalore-560065, India; 2 Current address: National Institute of Biomedical Innovation, 7-6-8 Asagi Saito Ibaraki-City, Osaka, 567-0085, Japan

## Abstract

**Background:**

Serine proteases are one of the most abundant groups of proteolytic enzymes found in all the kingdoms of life. While studies have established significant roles for many prokaryotic serine proteases in several physiological processes, such as those associated with metabolism, cell signalling, defense response and development, functional associations for a large number of prokaryotic serine proteases are relatively unknown. Current analysis is aimed at understanding the distribution and probable biological functions of the select serine proteases encoded in representative prokaryotic organisms.

**Results:**

A total of 966 putative serine proteases, belonging to five families, were identified in the 91 prokaryotic genomes using various sensitive sequence search techniques. Phylogenetic analysis reveals several species-specific clusters of serine proteases suggesting their possible involvement in organism-specific functions. Atypical phylogenetic associations suggest an important role for lateral gene transfer events in facilitating the widespread distribution of the serine proteases in the prokaryotes. Domain organisations of the gene products were analysed, employing sensitive sequence search methods, to infer their probable biological functions. Trypsin, subtilisin and Lon protease families account for a significant proportion of the multi-domain representatives, while the D-Ala-D-Ala carboxypeptidase and the Clp protease families are mostly single-domain polypeptides in prokaryotes. Regulatory domains for protein interaction, signalling, pathogenesis, cell adhesion *etc*. were found tethered to the serine protease domains. Some domain combinations (such as S1-PDZ; LON-AAA-S16 *etc*.) were found to be widespread in the prokaryotic lineages suggesting a critical role in prokaryotes.

**Conclusion:**

Domain architectures of many serine proteases and their homologues identified in prokaryotes are very different from those observed in eukaryotes, suggesting distinct roles for serine proteases in prokaryotes. Many domain combinations were found unique to specific prokaryotic species, suggesting functional specialisation in various cellular and physiological processes.

## Background

The proper functioning of a cell is facilitated by a precise regulation of protein levels, which in turn is maintained by a balance between the rates of protein synthesis and degradation. Protein degradation mediated by proteolysis is an important mechanism for recycling of the amino acids into the cellular pool and to possibly generate energy during starvation. Proteins like enzymes, transcription factors, receptors, structural proteins *etc*. require proteolytic processing for activation or functional changes. Proteolysis also contributes to the timely inactivation of proteins and is a major biological regulatory mechanism in living systems [1-4].

Serine proteases are ubiquitous enzymes with a nucleophilic Ser residue at the active site and believed to constitute nearly one-third of all the known proteolytic enzymes. They include exopeptidases and endopeptidases belonging to different protein families grouped into clans. Over 50 serine protease families are currently classified by MEROPS [5]. They function in diverse biological processes such as digestion, blood clotting, fertilisation, development, complement activation, pathogenesis, apoptosis, immune response, secondary metabolism, with imbalances causing diseases like arthritis and tumors [6-9]. Thus, many serine proteases and their substrates are attractive targets for therapeutic drug design.

Proteases play a significant role in adaptive responses of prokaryotes to changes in their extracellular environment by facilitating restructuring of their proteomes. Prokaryotic serine proteases are involved in several physiological processes associated with cell signalling, defense response and development [3,10,11]. DegP proteases belonging to the trypsin family have been implicated in heat shock response [12], subtilisins in growth and defense response in several bacteria [13], in nutrition and host invasion [14], serine β-lactamases in helping certain bacteria acquire resistance to β-lactam antibiotics [15] and Clp and Lon proteases in the removal of the misfolded proteins [16]. In addition, serine proteases are required for virulence in many pathogenic bacteria [17,18]. However, an understanding of the biological functions of large numbers of prokaryotic serine proteases remains elusive. A better understanding of their distribution and evolution in the prokaryotic lineages would help unravel their potential roles in the various cellular processes including pathogenesis and help develop effective antibacterial therapies. Therefore, five serine protease families- Trypsin (MEROPS S1), Subtilisins (MEROPS S8), DD-peptidases (MEROPS S12), Clp proteases (MEROPS S14) and Lon proteases (MEROPS S16), which have been implicated in diverse physiological processes in prokaryotes and represent some of the independent evolutionary lineages of the serine proteases were chosen as the model representatives for a genome-wide survey in select prokaryotic genomes.

The availability of the complete protein sequences of several bacterial and archaeal species makes it possible to carry out a comprehensive analysis to examine the complexity and the evolutionary relationships between the serine protease families and identify new proteolytic components in prokaryotes. Bioinformatics searches for the serine protease-like proteins belonging to the five serine protease families were performed in the 91 representative prokaryotic genomes (17 archaeal and 74 bacterial) for which complete genomic data are available, using various sensitive sequence search methods. Manual analysis was performed for serine proteases, identified above, to assess the presence or absence of key residues responsible for catalysis and substrate specificity. In several serine proteases, adjacent domains are often responsible for substrate specificity and/or involvement of serine proteases into specific physiological pathways. Therefore, the domain organisations of the putative serine proteases predicted based on the sequence similarity were analysed to understand their evolution and the probable biological roles.

## Methods

### Search for Serine Proteases in Prokaryotic Genomes

Complete proteomes for the 91 representative prokaryotic species were obtained from NCBI [19]. To facilitate the coverage of the serine protease repertoire in diverse genotypes, the proteomes of the select prokaryotes representing the different taxonomic lineages in prokaryotes and occupying diverse ecological niches were chosen for the analysis. These include extremophiles (such as *Aeropyrum pernix*- hyperthermophilic, *Halobacterium*- halophilic *etc*.); commercially significant microorganisms (*Corynebacterium efficiens*); pathogenic prokaryotes that infect bacteria, insects, plants, animals and humans (such as *Mycobacterium leprae*, *Agrobacterium tumefaciens*, *Bdellovibrio bacteriovorus*), symbiotic prokaryotes (*Azoarcus*; *Bradyrhizobium japonicum*), model organisms (*Synechocystis, Ralstonia eutropha*) *etc*. A search for serine proteases was performed using BLASTP [20] on the prokaryotic proteomes, using sequences for each serine protease family, as classified by MEROPS [5] for preliminary queries, complemented by a multi-fold approach that employs sensitive sequence search methods such as HMMPFAM [21] and RPS-BLAST [22] as described previously [23].

### Relative Densities of Distribution of Serine Proteases in Prokaryotic Genomes

The relative abundance of the five serine protease families in various prokaryotic lineages was also examined in the form of their relative densities. Relative density is defined here as the total number of serine proteases identified in a taxonomic lineage divided by the total number of genomes of that lineage considered for the study. Comparison of relative density values provides insights into the relative significance of the five serine protease families in different prokaryotic lineages, which in combination with the data from other sources such as phylogeny and domain architectures can provide useful insights into their probable functional associations.

### Identification of Co-existing Domains in Prokaryotic Serine Proteases

Co-existing domains were predicted using HMMPFAM of the HMMER suite [21]. Each serine protease sequence was matched to the dataset of Hidden Markov Models (HMMs) obtained from the PfamA database [24] with the E-value thresholds set to 0.1 [23]. Conservation of the domain architectures across the lineages was examined with NCBI-CDART [25] and Pfam [24].

### Multiple Sequence Alignment and Phylogenetic Analysis

Multiple sequence alignments of the serine protease domains were performed using CLUSTALW [26]; an overall phylogenetic tree was inferred from the multiple sequence alignment with PHYLIP (Phylogeny Inference Package) [27] and phylogenetic analysis was performed as described previously [23]. Representations of the calculated trees were constructed using MEGA program [28]. Clusters with bootstrap values greater than 50% were defined as confirmed subgroups and sequences with lower values added to these subgroups according to their sequence similarity in the alignment as judged by visual inspection.

## Results and discussion

### Distribution of the Five Serine Protease Families in the Prokaryotic Genomes

A total of 966 serine proteases belonging to the five serine protease families were identified in the 91 prokaryotic genomes for which the complete genomic data is available. These include 42 putative catalytically inactive serine protease homologues (hereafter uniformly referred to as SPHs) that either lack the amino acid residues essential for catalysis or carry amino acid substitutions at those positions. Such inactive enzyme homologues are present in many enzyme families and are believed to acquire newer functions during evolution [29,30] (Additional file 1). Trypsin (S1), subtilisin (S8) and D-Ala-D-Ala carboxypeptidase (DD-peptidase) (S12) families were found to have a higher number of representatives than Clp protease (S14) and Lon protease (S16) families (Table 1). The five serine protease families have a relatively lower representation in the archaeal genomes than the bacterial genomes. However, the relative densities (see Methods) of subtilisin and Lon protease families (S8 and S16 are 2.46 and 1.2, respectively) in archaeal genomes are much higher than other three families (0.33) (Table 2). The DD-peptidase family shows maximum abundance in the Alphaproteobacteria (relative density 7.1), while trypsins, subtilisins and Clp proteases have higher number of representatives in the Actinobacteria (relative densities 6.37, 4.1 and 2.37, respectively) and Lon proteases in the Gammaproteobacteria (relative density 2.4). Higher densities for trypsins, subtilisins and Lon proteases are observed in the Delatproteobacteria, largely due to only two Deltaproteobacteria genomes considered for the present analysis and the overrepresentation of the trypsins and the subtilisins in *Bdellovibrio_bacteriovorus*. High representations of some serine proteases were observed in other genomes such as *Streptomyces avermitilis*, indicating specific requirements (Tables 1, 2). Expansion of the specific protein families and/or superfamilies often occurs as a consequence of specialisation of an organism for its environmental niche and an investigation of the relative abundance of the specific protein families within the different prokaryotic species is likely to provide useful insights into their evolution and specialisation [31,32]. Thus, higher representation of the trypsin, subtilisin and the DD-peptidase families suggests the evolution of specialised functions for the gene products corresponding to the three families in many representative species chosen for the present analysis (Additional file 2). Putative serine proteases thus, identified, were further analysed for the presence of the co-existing domains. The occurrence, the domain organisation and the phylogenetic patterns of the select serine protease domains in the specific prokaryotic genomes are discussed below.

**Table 1:** Distribution of the select serine protease families across the representative genomes of the prokaryotic lineages.

**Prokaryotic Genomes**	**Tryp**	**Subt**	**DDpept**	**Clp**	**Lon**
**Archaea**					

**Crenarchaeota**					

*Aeropyrum_pernix *(Chromosome)	-	3	1	2	-

*Pyrobaculum_aerophilum *(Chromosome)	1	2	1	1	-

*Sulfolobus_acidocaldarius_DSM_639 *(Chromosome)	2	5	-	-	-

*Sulfolobus_solfataricus *(Chromosome)	1	2	-	-	-

*Sulfolobus_tokodaii *(Chromosome)	1	3	-	-	-

					

**Euryarchaeota**					

*Archaeoglobus_fulgidus *(Chromosome)	-	2	-	-	2

*Halobacterium_sp_NRC1 *(1Chromosome + 2 plasmids)	1	2	-	-	1

*Methanococcoides_burtonii_DSM_6 *(Chromosome)	-	2	-	-	1

*Methanococcus_jannaschii *(3Chromosomes)	-	-	-	-	2

*Methanosarcina_acetivorans *(Chromosome)	-	3	1	-	2

*Methanosarcina_mazei *(Chromosome)	-	2	1	-	2

*Natronomonas_pharaonis *(Chromosome + 2 plasmids)	1	3	-	-	1

*Pyrococcus_abyssi *(Chromosome + 1 plasmid)	-	-	1	1	2

*Pyrococcus_furiosus *(Chromosome)	-	3	-	1	2

*Thermococcus_kodakaraensis_KOD1 *(Chromosome)	-	3	-	-	2

*Thermoplasma_acidophilum *(Chromosome)	-	2	-	-	1

					

**Nanoarchaeota**					

*Nanoarchaeum_equitans *(Chromosome)	-	-	-	-	1

**Bacteria**					

**Actinobacteria**					

*Corynebacterium_efficiens_YS-314 *(Chromosome)	7	1	2	2	-

*Corynebacterium_glutamicum_ATCC_13032_Bielefeld *(Chromosome)	7	1	2	2	-

*Mycobacterium_leprae *(Chromosome)	4	3	2	2	1

*Mycobacterium_tuberculosis_CDC1551 *(Chromosome)	6	4	13	2	1

*Propionibacterium_acnes_KPA17120 *(Chromosome)	2	1	2	2	1

*Rubrobacter_xylanophilus_DSM_9941*(Chromosome)	7	3	3	1	1

*Streptomyces_avermitilis *(1Chromosome + 1 plasmid)	14	15	13	5	2

*Symbiobacterium_thermophilum_IAM14863 *(Chromosome)	4	5	9	3	3

					

**Alphaproteobacteria**					

*Agrobacterium_tumefaciens_C58_Cereon *(2 Chromosomes + 2 plasmids)	3	-	3	3	1

*Bradyrhizobium_japonicum *(Chromosome)	10	2	17	2	2

*Mesorhizobium_loti *(1Chromosome + 2 plasmids)	8	2	7	3	1

*Novosphingobium_aromaticivorans_DSM_12444 *(1 Chromosome + 2 plasmids)	2	2	7	1	1

*Rhodopseudomonas_palustris_CGA009 *(1 Chromosome +1 plasmid)	8	2	7	1	1

*Sinorhizobium_meliloti *(1 Chromosome +2 plasmids)	1	2	2	-	-

					

**Betaproteobacteria**					

*Azoarcus_sp_EbN1 *(Chromosome)	4	3	-	2	3

*Bordetella_bronchiseptica *(Chromosome)	2	2	2	2	2

*Bordetella_parapertussis *(Chromosome)	2	1	2	1	2

*Burkholderia_cenocepacia_AU_1054 *(3 Chromosomes)	3	1	1	-	-

*Burkholderia_mallei_ATCC_23344 *(2 Chromosomes)	3	2	1	1	1

*Burkholderia_thailandensis_E264 *(2 Chromosomes)	-	6	3	1	1

*Chromobacterium_violaceum *(Chromosome)	6	4	2	2	1

*Neisseria_meningitidis_MC58 *(Chromosome)	1	1	-	1	1

*Ralstonia_eutropha_JMP134 *(2 Chromosomes + 2 plasmids)	-	1	1	-	-

*Ralstonia_solanacearum *(1 Chromosome + 1 plasmid)	3	3	1	1	1

					

**Chlorobi**					

*Bacteroides_thetaiotaomicron_VPI-5482 *(1 Chromosome + 1 plasmid)	3	2	2	1	1

*Pelodictyon_luteolum_DSM_273 *(Chromosome)	3	1	1	1	1

*Salinibacter_ruber_DSM_13855 *(1 Chromosome + 1 plasmid)	2	5	4	2	-

					

**Cyanobacteria**					

*Gloeobacter_violaceus *(Chromosome)	7	4	4	2	-

*Synechococcus_CC9605 *(Chromosome)	3	1	3	3	-

*Synechocystis_PCC6803 *(1 Chromosome + 4 plasmids)	3	1	3	4	-

					

**Deinococcus-Thermus**					

*Deinococcus_radiodurans *(2 Chromosomes + 2 plasmids)	-	1	-	-	-

					

**Deltaproteobacteria**					

*Bdellovibrio_bacteriovorus *(Chromosome)	24	15	2	1	3

*Geobacter_sulfurreducens *(Chromosome)	2	3	2	1	5

					

**Epsilonproteobacteria**					

*Thiomicrospira_crunogena_XCL-2 *(Chromosome)	1	1	2	1	3

					

**Firmicutes**					

*Bacillus_anthracis_Ames *(Chromosome)	2	4	14	4	3

*Bacillus_clausii_KSM-K16 *(Chromosome)	2	5	4	3	3

*Bacillus_halodurans *(Chromosome)	2	9	2	3	3

*Bacillus_subtilis *(Chromosome)	4	7	4	2	3

*Bacillus_thuringiensis_konkukian *(1 Chromosome + 1 plasmid)	2	5	17	3	3

*Enterococcus_faecalis_V583 *(1 Chromosome + 3 plasmids)	2	-	3	1	-

*Lactobacillus_acidophilus_NCFM *(Chromosome)	1	1	6	1	1

*Lactobacillus_johnsonii_NCC_533 *(Chromosome)	1	1	3	1	1

*Lactobacillus_sakei_23K *(Chromosome)	1	-	3	1	-

*Lactococcus_lactis *(Chromosome)	1	-	1	1	1

*Oceanobacillus_iheyensis *(Chromosome)	3	8	4	2	1

*Staphylococcus_aureus_COL *(Chromosome)	9	1	2	2	-

*Staphylococcus_epidermidis_ATCC_12228 *(1 Chromosome + 1 plasmid)	3	-	3	1	-

*Streptococcus_agalactiae_2603 *(Chromosome)	1	3	2	1	-

*Streptococcus_mutans *(Chromosome)	1	-	2	1	-

*Streptococcus_pneumoniae_TIGR4 *(Chromosome)	2	1	1	1	-

*Thermoanaerobacter_tengcongensis *(Chromosome)	2	3	2	2	2

*Thermus_thermophilus_HB27 *(1 Chromosome + 1 plasmid)	3	1	2	1	3

					

**Fusobacteria**					

*Fusobacterium_nucleatum *(Chromosome)	1	2	-	1	1

					

**Gammaproteobacteria**					

*Escherichia_coli_O157H7_EDL933 *(1 Chromosome + 1 plasmid)	4	-	3	4	2

*Haemophilus_influenzae *(Chromosome)	2	-	-	1	2

*Hahella_chejuensis_KCTC_2396 *(Chromosome)	8	4	4	2	3

*Idiomarina_loihiensis_L2TR *(Chromosome)	2	2	3	1	2

*Photorhabdus_luminescens *(Chromosome)	3	3	7	1	1

*Pseudoalteromonas_haloplanktis_TAC125 *(2 Chromosomes)	2	2	3	1	2

*Pseudomonas_aeruginosa *(Chromosome)	2	2	7	3	3

*Pseudomonas_fluorescens_Pf-5 *(Chromosome)	2	2	7	3	4

*Pseudomonas_putida_KT2440 *(Chromosome)	2	2	4	3	4

*Pseudomonas_syringae_phaseolicola_1448A *(1 Chromosome + 2 plasmids)	3	2	4	1	4

*Shewanella_oneidensis *(1 Chromosome + 1 plasmid)	2	6	2	2	2

*Xanthomonas_campestris *(Chromosome)	3	10	3	1	1

*Xylella_fastidiosa *(Chromosome)	2	3	1	2	1

					

**Spirochaetes**					

*Treponema_denticola_ATCC_35405 *(Chromosome)	5	-	1	2	1

					

**Total**	**247**	**227**	**254**	**121**	**117**

Tryp- Trypsin (Pfam accession – PF00089); Subt- Subtilisin (PF00082); DDPept- D-Ala-D-Ala carboxypeptidase B (DD-peptidase) (PF00144); Clp- ClP protease (PF00574); Lon- Lon protease (PF05362).

Genomes where no serine protease-like proteins were identified: *Bacteroides_fragilis_YCH46 Haloarcula_marismortui_ATCC_43049*; *Legionella_pneumophila_Lens*; *Nostoc_sp*.; *Salmonella_enterica_Choleraesuis*; *Clostridium_acetobutylicum*; *Clostridium_perfringens*

**Table 2:** Distribution of five serine protease families across various prokaryotic taxonomic groups represented in 91 genomes.

Lineage	Trypsin	Subtilisin	Beta-lactamase	Clp protease	Lon protease
Euryarchaeota (11)	2	22	3	2	18

Crenarchaeota (5)	5	15	2	3	-

Alphaproteobacteria (6)	32	10	43	10	6

Betaproteobacteria (10)	24	24	13	11	12

Gammaproteobacteria (13)	37	38	48	25	31

Firmicutes (18)	42	49	75	31	24

Actinobacteria (8)	51	33	46	19	9

Chlorobi (3)	8	8	7	4	2

Cyanobacteria (3)	13	6	10	9	-

Deltaproteobacteria (2)	26	18	4	2	8

Others (12)	7	4	3	15	7

Total	247	227	254	121	117

### Trypsin (S1 Family)

Trypsins (or S1 proteases) constitute the largest group of proteolytic enzymes that display diverse specificities and function as endopeptidases. Their catalytic apparatus consists of the conserved Asp(102)-His(57)-Ser(195) "charge relay" system, called the catalytic triad. This triad was initially identified in trypsin-like serine proteases and later found in the other distinct folds such as subtilisins, serine carboxypeptidases and Clp proteases, though the catalytic residues occur in a different order in their sequences: a typical example of convergent evolution of the same biochemical mechanism in structurally distinct folds [6,33,34]. Broadly, three main activity types for cleavage of amide substrates have been recognised for this enzyme family: trypsin-like enzymes show overwhelming preference for Arg/Lys at the P1 site (first substrate amino acid N-terminal to the scissile bond), chymotrypsin-like enzymes prefer aromatic amino acids at the P1 position, while elastase-like enzymes prefer substrates with small hydrophobic amino acids at P1 positions [5]. In prokaryotes, trypsin-like serine proteases function in diverse processes such as bacterial cell wall lysis [35], heat shock response [12], transcription regulation [36], as toxins [37], as fibrinolytic enzymes [38]*etc*.

A total of 247 putative trypsin-like serine proteases (2 SPHs) were identified in the 91 prokaryotic genomes under study (Additional file 1). They are well-represented in all the prokaryotic lineages considered in the present analyses, though a low representation was observed in archaeal genomes (Tables 1, 2). Over half of the trypsin-like proteins identified in the present study were found to be multi-domain polypeptides. A significant number of the eukaryotic trypsin-like serine proteases have been found to carry accessory domains, which are believed to contribute to their functional diversity [23,30,39]. This suggests that the ancillary domains in the prokaryotic trypsin homologues may contribute to diversification in their function. Distribution of the trypsin-like serine proteases was not uniform across the genomes considered here (Tables 1, 2). Their over-representation in some species is probably a consequence of the organisms' adaptation to their environment. For instance, the highest numbers of trypsin-like proteins were identified in *Bdellovibrio bacteriovorus *(a highly motile Gram-negative bacterium) that preys on the other Gram-negative bacteria, which include plant, animal and human pathogens. The bacterium employs an extensive array of hydrolytic enzymes to invade its prey and consume the host biopolymers such as proteins. Proteases constitute the largest group of such paralogous hydrolytic enzymes, strongly suggesting their significant contribution to the life cycle of the bacterium [40]. The present analysis reveals a high abundance of the trypsin-like proteins in *Bdellovibrio bacteriovorus *(24 gene products; Table 1). Considering that trypsin-like serine proteases (such as alpha-lytic protease) are known to function in bacterial cell wall lysis [35], it is likely that many trypsin-like proteins in *Bdellovibrio bacteriovorus *may be associated with its predatory activities. Since all but one of these trypsin-like proteins are single-domain proteins (Additional file 1), it is likely that a precise and timely regulation of gene expression patterns may play a major role in regulating their activity [40].

**Figure 1:**
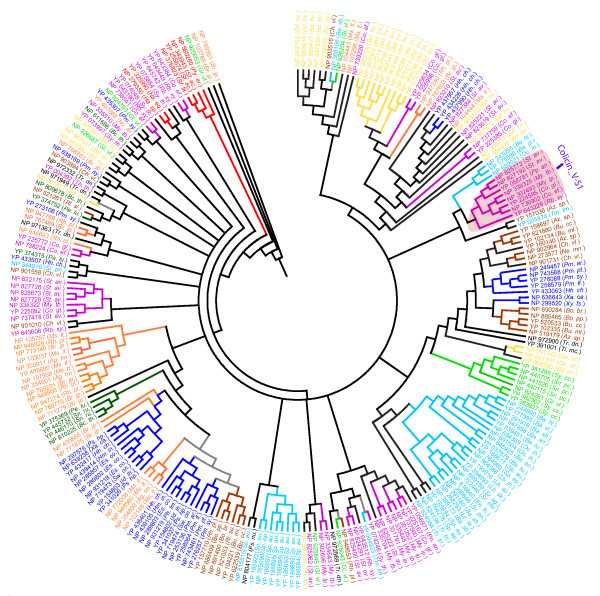
**Phylogenetic analysis of the trypsins.** A neighbour-joining tree based on an alignment of the trypsin protease domain generated with ClustalW [26], was inferred using the PHYLIP package [27] and drawn using the MEGA program [28] (see text for details). The various taxonomic lineages encountered in the analysis are represented in the different colours. For clarity, the protein identifiers are suffixed with the abbreviated species IDs (see Additional file 2). Only the protein clusters supported by significant bootstrap values (> 50%) are highlighted with the colour scheme. For the rest only the gene (and species) identifiers are highlighted with the colour scheme. The primary branches in the clusters populated by the representatives from non-identical lineage (taxa) are shaded in grey. Atypical members in an otherwise strong cluster are highlighted in the colour of their corresponding lineage. The phylogenetic clade corresponding to the trypsin-like proteins that carry the Colicin_V-S1 domain architecture is shaded pink. The colour schemes for the various lineages are as follows: Actinobacteria- Magenta; Alphaproteobacteria- Orange; Archaea- Red; Betaproteobacteria- Brown; Chlorobi- Olive green; Cyanobacteria- Green; Deltaproteobacteria- Yellow; Firmicutes- Cyan; Gammaprot- Gammaproteobacteria- Blue; Others- Black.

Phylogenetic analysis reveals the presence of several taxa-specific and species-specific clusters of the trypsin-like proteins in the prokaryotes (Figure 1). Trypsin-like proteins identified in *Bdellovibrio bacteriovorus *were found to cluster into four major groups (Figure 1). *Bdellovibrio bacteriovorus *deploys its hydrolytic arsenal at three distinct stages of its lifecycle [40], therefore, it is possible that various lineages of *Bdellovibrio bacteriovorus *trypsin-like proteins identified here may represent putative components of the hydrolytic machinery that drives the bacterium's predatory lifestyle. This makes them an attractive target for further characterisation, which may help to understand their mechanism of action better and aid in the development of new anti-microbial strategies. Trypsin-like proteins accompanied by Colicin_V domains, which are believed to function in pathogenesis, were observed only in the Gram-positive bacteria (Actinobacteria). In the phylogenetic trees constructed with the protease domains alone, all the trypsin-like proteins associated with a Colicin_V domain fall into a single cluster (Figure 1). Since co-existing domains in a multi-domain protein are often known to spatially interact with each other, the interface regions in the trypsin protease domain may acquire a pattern uniquely different from the homologous domains. Phylogenetic analysis also reveals clusters of trypsin-like proteins populated by members from different taxa, providing important clues to the evolution of this gene family in prokaryotes. For instance, while the trypsin-like protein NP_924281.1 from *Gloeobacter violaceus *co-clusters with the other trypsin-like proteins from cyanobacteria (labelled green), the other trypsin-like proteins from the same genome co-cluster with trypsin-like proteins from other taxa. For example, NP_926204.1 co-clusters with NP_388106.1 from *Bacillus subtilis *(Firmicutes), while NP_925645.1 co-clusters with NP_948653.1 from *Rhodopseudomonas palustris *(Alphaproteobacteria). Such occurrence may indicate a putative horizontal gene transfer of some trypsin-like proteins between *Gloeobacter violaceus*, a cyanobacterium and the other taxa (Figure 1). Indeed, horizontal gene transfer events have been documented between cyanobacteria and other phyla [41]. The abundance of the trypsin-like proteins in the prokaryotes may have partly resulted from the multiple horizontal gene transfer events between different species.

### Subtilisin (S8 Family)

Subtilisins constitute the second largest family of serine proteases identified till date and known members span across eubacteria, archaebacteria, eukaryotes and viruses. Subtilisins utilise a highly conserved catalytic triad similar to the members of the trypsin family, but have a different order of the Asp, His and Ser residues in the sequence (D137, H168, S325). Most members of the family exhibit broad substrate specificity, with a preference to cleave after the hydrophobic residues; however, some members of the S8B subfamily cleave peptide bonds just after dibasic amino acids [42]. Subtilisins in prokaryotes function in diverse processes such as cellular nutrition and host invasion [14], facilitating the maturation of diverse polypeptides [43] such as bacteriocins like lantibiotics [44], extracellular adhesins [45], enzymes such as the spore cortex-lytic enzyme in *Clostridium perfringens *[46]*etc*. Most known subtilisins are multi-domain polypeptides that consist of a protease domain accompanied by one or two co-existing domains [23], which also accounts for the diversity in their function.

**Figure 2:**
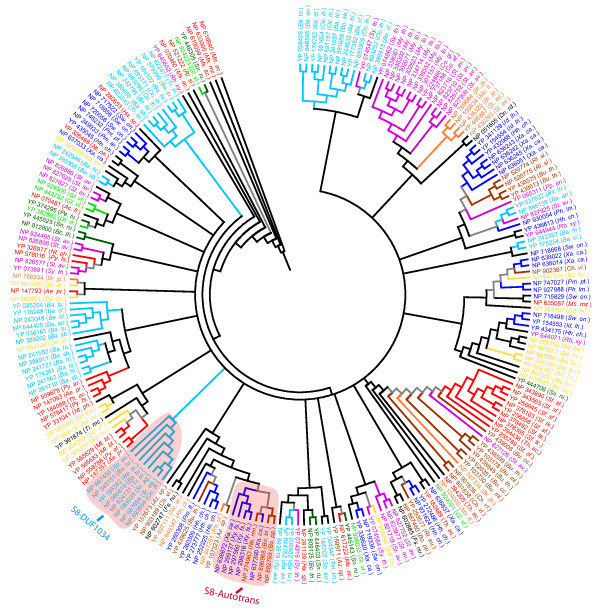
**Phylogenetic analysis of the subtilisins carried out as described in Figure 1.** Phylogenetic clade corresponding to subtilisin homologues that carry S8-Autotrans domain architecture and those that atleast carry a DUF1034 module C-terminal to subtilisin protease domain are marked. The abbreviations and the colour schemes are the same as in Figure 1.

A total of 227 subtilisin-like proteins (8 SPHs) were identified in the present study (Additional file 1). They are well represented in all the prokaryotic lineages suggesting that the subtilisin repertoires were established early in evolution (Tables 1, 2). A significant number of subtilisin-like proteins were identified in *Bdellovibrio bacteriovorus*, a predatory Gram-negative bacterium (15 gene products; Table 1) were found to fall into distinct clusters (Figure 2). Since prokaryotic subtilisins are known to function in the physiological processes associated with pathogenesis such as host invasion [14], it is likely that some subtilisin-like proteins may function as the specific components of the hydrolytic machinery employed by *Bdellovibrio bacteriovorus *for predation on the other Gram-negative bacteria [40]; the presence of the co-existing domains adjacent to the protease domain in many of these subtilisin-like proteins may also influence their involvement in different pathways, which in turn may regulate the predatory lifestyle of the bacterium (Table 3; Additional file 1). A significant number of the subtilisin-like proteins were also identified in *Streptomyces avermitilis *(15 gene products; Table 1), a commercially important Gram-positive soil bacterium known for its diversity in the production of the secondary metabolites. To facilitate this diversity, the bacterium contains several metabolic pathways for the biosynthesis of the secondary metabolites [47]. Subtilisins are known to function as the maturation proteases for several enzymes [43], and may thus, regulate the components of various metabolic pathways in *Streptomyces avermitilis*. Subtilisins are also known to function as maturation enzymes for the bacteriocin-like lantibiotics [44], which are peptide antibiotics produced by the Gram-positive bacteria [48]. It is likely that some subtilisins may process similar peptide antibiotics synthesised in *Streptomyces avermitilis*, thereby regulating their activity. Co-existing domains, associated with some subtilisin-like proteins in *Streptomyces avermitilis *(Table 3, Additional file 1), may facilitate the involvement these gene products for a regulatory role in the different metabolic pathways or for the recognition and processing (or even degradation) of the various secondary metabolites. However, for the other single domain proteins (Additional file 1), a precise and timely regulation of their transcription may play a major role in regulating their activity.

**Table 3:** Distribution of domain architectures in prokaryotic SPs; their occurrence in major lineages (indicated by +) and inferred functional associations based on co-existing domains and literature.

			Lineage*			
				
Domain Architecture^#^	Representative sequence	No. of SPs	A	B	E	Postulated Biological Functional Associations (see text)
Trypsin family (S1; Tryp(sin)- PF00089)						

Tryp	YP_643608.1	112	+	+	+	Proteolysis

Tryp-PDZ	NP_441326.1	63	+	+	+	Signalling

Tryp-PDZ-PDZ	NP_107958.1	49	-	+	-	Signalling, Heat Shock response

Colicin_V-Tryp	NP_338325.1	8	-	+	-	Pathogenesis, Defense

Pro_Al_prot-Tryp	NP_827728.1	2	-	+	-	Proteolysis

Tryp-Endonuclease_NS	NP_604177.1	1	-	+	-	Nucleic acid metabolism

Tryp-(FG-GAP)_3_	NP_825221.1	1	-	+	-	Ligand binding and processing

Tryp-(Sel1)_6_	YP_374752.1	1	-	+	-	Proteolysis

Tryp-CW_binding_1-CW_binding_1	NP_344916.1	1	-	+	-	Cell recognition, pathogenesis

FHA-FHA-Tryp	NP_811686.1	1	-	+	-	Metabolism and signalling

Pro_Al_prot-Tryp-CBM_5_12	NP_822175.1	1	-	+	-	Carbohydrate metabolism

(Pro_Al_prot)_2_-Tryp	NP_827729.1	1	-	+	-	Proteolysis

Tryp-ANF_receptor	YP_073997.1	1	-	+	-	Ligand binding and processing

Tryp-PPC-SCP	YP_434226.1	1	-	+	-	Calcium chelating, signalling

Tryp-PPC-PPC	YP_437990.1	1	-	+	-	Carbohydrate metabolism, signalling

TerD-Tryp	YP_273108.1	1	-	+	-	Growth in unfavourable environment

Subtilisin family (S8; Subt(ilisin)- PF00082)						

Subt	NP_147093.1	142	+	+	+	Proteolysis

Subt-Autotransporter	YP_260308.1	15	-	+	-	Transport, Cell adhesion, Virulence

Subtilisin_N-Subt	NP_241550.1	14	+	+	+	Proteolysis

Subt-PPC	YP_154554.1	9	+	+	-	Carbohydrate metabolism, signalling

Subtilisin_N-Subt-PA	NP_391688.1	4	+	+	+	Proteolysis

Subt-PPC-PPC	YP_341139.1	4	+	+	-	Carbohydrate metabolism, signalling

Subt-P_proprotein	NP_967370.1	3	-	+	-	Proteolysis

Subt-Big_2	NP_969490.1	2	-	+	-	Cell adhesion, pathogenesis

Subt-DUF1034	YP_194362.1	2	-	+	-	Proteolysis

Subt-PA-DUF1034	NP_693854.1	2	-	+	+	Proteolysis

Subt-PKD-PKD	YP_326498.1	2	+	+	+	Carbohydrate metabolism, signalling

Subt-P_proprotein-PKD	NP_716498.1	2	-	+	-	Carbohydrate metabolism, signalling

GRP-Subt	NP_435320.1	1	-	+	-	Stress response

(Hemolys)_2_-Subt-P_proprotein	NP_747027.1	1	-	+	-	Cell surface binding

(Hemolys)_3_-Subt-P_proprot-Hemolys	NP_927988.1	1	-	+	-	Cell surface binding

PPC-Subt	YP_436813.1	1	-	+	-	Carbohydrate metabolism, signalling

Subt-BNR	NP_824495.1	1	-	+	-	Proteolysis

Subt-(CARDB)_9_	NP_954260.1	1	-	+	-	Cell adhesion, pathogenesis

Subt-Cleaved_Adhesin-fn3-PKD-PKD	YP_074547.1	1	-	+	-	Virulence, signalling, metabolism

Subt-CUB	NP_967057.1	1	-	+	+	Signalling

Subt-(Dockerin_1)_2_	NP_280653.1	1	-	+	-	Cellulose degradation, metabolism

Subt-DUF11	NP_951948.1	1	-	+	-	Cellular transport

Subt-fn3	YP_446403.1	1	-	+	-	Cell surface binding

Subt-(fn3)_3_-(PKD)_3_	YP_565583.1	1	-	+	-	Cell surface binding, signalling, metabolism

Subt-Gram_pos_anchor	NP_241562.1	1	-	+	-	Cell invasion, pathogenesis

Subt-NosD	NP_616940.1	1	+	-	-	Respiratory metabolism

Subt-PA-DUF1034-(Big_2)_2_-(SLH)_2_	NP_624131.1	1	-	+	-	Cell adhesion, pathogenesis

Subt-PilZ	NP_969350.1	1	-	+	-	Signalling

Subt-(P_proprotein)_2_	YP_434175.1	1	-	+	-	Proteolysis

Sub_N-Subt-Cleaved_Adhesin	NP_693252.1	1	-	+	-	Virulence

Sub_N-Subt-PA-Dockerin	NP_691157.1	1	-	+	-	Cellulose degradation, metabolism

Sub_N-Subt-PA-DUF1034-Gram_pos_anchor	NP_345151.1	1	-	+	-	Cell invasion, pathogenesis

Sub_N-Subt-PA-DUF1034-(FIVAR)_5_-Gram_pos_anchor	NP_965819.1	1	-	+	-	Cell recognition and invasion, Sugar binding

Sub_N-Subt-PA-PPC	NP_717522.1	1	-	+	-	Carbohydrate metabolism, signalling

Sub_N-Subt-PA-PPC- P_proprotein	NP_718668.1	1	-	+	-	Carbohydrate metabolism, signalling

Thermopsin-Subt	NP_394205.1	1	+	-	-	Thermostability

(W_rich_C)_2_-(PPC)_2_-Subt	YP_382882.1	1	-	+	-	Cell surface signalling

YSIRK_signal-Subt-PA-DUF1034-(FIVAR)_3_- Gram_pos_anchor	NP_689039.1	1	-	+	-	Cell recognition and invasion, Sugar binding

DD-peptidase family (S12; DD-Pept(idase) -PF00144)						

DDPept	NP_811352.1	249	+	+	+	Cell wall biosynthesis

DDPept -ABC_tran	YP_434618.1	1	-	+	-	Biological transport

DDPept -DUF1343	YP_439122.1	1	-	+	-	Cell wall biosynthesis

(Cond-AMP-PPbind)_3_- DDPept	NP_824819.1	1	-	+	-	Metabolism of Antibiotic compounds

Glyco_hydr_3- DDPept	NP_811352.1	1	-	+	-	Carbohydrate hydrolysis

Glyc_hyd_3_Glyc_hyd_3_C- DDPept	YP_444518.1	1	-	+	-	Carbohydrate hydrolysis, metabolism

Clp protease family (S14; Clp(_protease)- PF00574)						

Clp	NP_811352.1	118	+	+	+	Proteolysis

Clp-Nfed	NP_126341.1	3	+	+	-	Proteolysis

Lon protease family (S16; Lon_C- PF05362)						

Lon_C	NP_623361.1	39	+	+	+	Proteolysis

LON-AAA-Lon_C	NP_743601.1	58	+	+	+	Signalling, metabolism

Sigma54_activat-AAA-Lon_C	YP_183677.1	8	+	+	-	Transcription regulation, metabolism

Sigma54_activat-Lon_C	NP_127256.1	5	+	-	-	Transcription regulation

Mg_chelatase-Lon_C	NP_248420.1	4	+	-	-	Bacteriochlorophyll metabolism

Mg_chelat-Sigma54_activat-Lon_C	NP_578196.1	1	+	-	-	Transcription regulation

DnaB_C-Tryp	YP_160730.1	1	-	+	-	DNA metabolism

PDZ-Lon_C	NP_389388.1	1	-	+	-	Signalling

# Co-exisiting domains: **Tryp**-Trypsin; **Subt**-Subtilisin; **DDPept**- DD-peptidase; **Clp**- Clp protease; **Lon**_**C**- Lon protease; **AAA**- ATPase family associated with various cellular activities (PF00004); **ABC_tran**- ABC transporter (PF00005); **AMP-binding**- AMP-binding enzyme (PF00501); **ANF_receptor**- Receptor family ligand binding region (PF01094); Autotransporter- Autotransporter beta-domain (PF03797); **Big_2**- Bacterial Ig-like domain (group 2) (PF02368); **BNR**- BNR/Asp-box repeat (PF02012); **CARDB**- Cell adhesion related domain found in bacteria (PF07705); **Colicin_V**- Colicin V production protein (PF02674); **CBM_5_12**- Carbohydrate binding domain (PF02839); **Cleaved_Adhesin**- Cleaved Adhesin Domain (PF07675); **Cond(ensation)**- Condensation domain (PF00668); **CUB**- CUB domain (PF00431); **CW_binding_1**- Putative cell wall binding repeat (PF01473); **DnaB_C**- DnaB-like helicase C terminal domain (PF03796); Dockerin_1- Dockerin type I repeat (PF00404); **DUF1034**- Domain of unknown function (PF06280); **DUF11**- Domain of unknown function (PF01345); **DUF1343**- Protein of unknown function (PF07075); **FG-GAP**- FG-GAP repeat (PF01839); **Endonuclease_NS**- DNA/RNA non-specific endonuclease (PF01233); **FHA**- FHA (Forkhead-associated) domain (PF00498); **FIVAR**- Uncharacterised Sugar-binding Domain (PF07554); **fn3**- Fibronectin type III domain (PF00041); **Glyc_hyd_3**- Glycosyl hydrolase family 3 N terminal domain (PF00933); **Glyc_hydr_3_C**- Glycosyl hydrolase family 3 C terminal domain (PF01915); **Gram_pos_anchor**- Gram positive anchor (PF00746); **GRP**- Glycine rich protein family (PF07172); **Hemolys**- Hemolysin-type calcium-binding repeat (2 copies) (PF00353); **LON**- ATP-dependent protease La (LON) domain (PF02190); **Mg_chelat**- Magnesium chelatase, subunit ChlI (PF01078); **Nfed**- Nfed-like (PF01957); **NosD**- Periplasmic copper-binding protein (NosD) (PF05048); **P_proprotein**- Proprotein convertase P-domain (PF01483); **PA**- Protease associated domain (PF02225); **PDZ**- PDZ domain (PF00595); **PKD**- PKD domain (PF00801); **PP-binding**- Phosphopantetheine attachment site (PF00550); **PPC**- Bacterial pre-peptidase C-terminal domain (PF04151); **Pro_Al_prot**- Alpha-lytic protease prodomain (PF02983); **Sel**1- Sel1 repeat (PF08238); **SCP**- SCP-like extracellular protein (PF00188); **Sigma54_activat**- Sigma-54 interaction domain (PF00158); **SLH**- S-layer homology domain (PF00395); **Sub(tilisin)_N**- Subtilisin N-terminal region (PF005922); **TerD**- Bacterial stress protein (PF02342); **Thermopsin**- Thermopsin (PF05317); **W_rich_C**- Tryptophan-rich Synechocystis species C-terminal domain (PF07483) * Lineage: A- Archaea; B- Bacteria; E- Eukaryotes

Phylogenetic analysis reveals the presence of several taxa-specific and species-specific clusters of subtilisin-like proteins (Figure 2). Subtilisin-like proteins identified in *Bdellovibrio bacteriovorus *were found to fall into different clusters that may correspond to gene products associated with the predatory machinery of the bacterium (Figure 2). Five major clusters of the subtilisin-like proteins in *Streptomyces avermitilis *were also recognised, indicating the evolutionary and possibly the functional diversity of these gene products in the bacterium (Figure 2). Like trypsins, some subtilisin-like proteins associated with the specific co-existing modules cluster together in the phylogenetic tree constructed with the subtilisin protease domain sequences alone. The subtilisin-like proteins from Betaproteobacteria and Gammaproteobacteria that are associated with the autotransporter modules were observed to cluster together (Figure 2). Many subtilisin-like proteins from the Gram-positive bacteria (Firmicutes) that at least carry a DUF1034 module, C-terminal to the predicted protease domain, cluster together (Figure 2). The probable spatial interactions between the subtilisin protease domain and the adjacent modules may result in the acquisition of unique or differential patterns in the interface region of the protease domain. Phylogenetic analysis also reveals some associations between subtilisins identified in different prokaryotic species that suggest probable horizontal transfer of subtilisin genes between prokaryotes. The co-clustering of a subtilisin-autotransporter gene product NP_637390.1 (*Xanthomonas campestris*) with similar proteins from betaproteobacteria i.e. NP_274963.1 (*Neisseria meningitides MC58*); NP_886968.1 (*Bordetella bronchiseptica*) and NP_882769 (*Bordetella parapertussis*) instead of the gammaproteobacteria, suggests a lateral acquisition of the former from the betaproteobacteria. Archaeal subtilisins YP_326977.1 (*Natronomonas pharaonis*) and NP_578046.1 (*Pyrococcus furiosus*) co-cluster with NP_826577.1 (*Streptomyces avermitilis*) and YP_073891.1 (*Symbiobacterium thermophilum IAM14863*), while YP_565629.1 and YP_565583.1 (*Methanococcoides burtonii*), NP_147357.1 (*Aeropyrum pernix*) and NP_558788.1 (*Pyrobaculum aerophilum*) co-cluster with NP_969150.1 (*Bdellovibrio bacteriovorus*) and NP_954260.1 (*Geobacter- sulfurreducens*) suggesting a lateral transfer of some subtilisin genes between bacteria and archaea. It is possible that the abundance of the subtilisins in the prokaryotes may have been facilitated in part by multiple horizontal gene transfer events.

### D-Ala-D-Ala carboxypeptidase B Family (S12)

The D-Ala-D-Ala carboxypeptidase B (DD-peptidase) family is a diverse family that consists of proteins performing varied functions such as D-Alanyl-D-alanine carboxypeptidase B (DD-peptidase), aminopeptidase (DmpB), class A, C β-lactamases *etc*. DD-peptidases (Penicillin binding proteins/PBPs) are β-lactam sensitive enzymes that process precursor peptides that facilitate peptidoglycan cross-linking during bacterial cell wall biosynthesis [49]. Studies have led to the identification of their active site residues (S93, K96, Y190). The active site Ser and Lys residues form a sequential motif that is highly conserved across family members. The Tyr active site residue occurs in a conserved Y-x-N motif situated on a loop in the all α-domain, with Tyr residue being replaced by Ser in some proteins [15,50,51]. β-lactamases are hydrolases that catalyse the hydrolysis of the β-lactam ring of β-lactam antibiotics such as penicillins. and probably evolved as a means of protection against the β-lactam antibiotics that restrict the sacular growth (peptidoglycan biosynthesis) in bacteria by inhibiting DD-peptidase activity [15,50,52]. Class A (penicillinase type) proteins were the first to be identified and are the most common β-lactamases. DD-peptidase-like proteins are widespread in the bacterial genomes, and their corresponding genes may occur on bacterial chromosomes or on plasmids. This allows for their transfer to the distant species and may account for their distribution and diversity [51-53]. The absence of DD-peptidase-like proteins in most eukaryotic lineages is attributed to the absence of peptidoglycans especially in metazoa [54,55].

A total of 254 DD-peptidase-like proteins (9 SPHs) were identified in the current analysis (Tables 1, 2; Additional file 1). While they display a widespread distribution in the bacteria, a low representation is observed for these enzymes in the archaeal genomes considered in the present study (Table 1, Additional file 1). This is probably due to the different pathways for cell wall biosynthesis in archaea which involve pseudomureins. The abundance of the DD-peptidase-like proteins in bacterial lineages is attributed to their ancient evolution as important constituents of the cell wall biosynthesis in bacteria, specialisation of a significant repertoire as β-lactamases for protection against the β-lactam compounds and their retention in adaptation to probable subsequent modifications in the β-lactam synthesising pathways that share the ACV synthetase gene, which is widely distributed in the bacterial genomes [15]. A closer inspection of the distribution of the DD-peptidase-like proteins in the prokaryotic species considered in the present study reveals a high representation in the genomes of some pathogenic bacteria: *Bacillus anthracis Ames *(14 gene products), *Bacillus thuringiensis konkukian *(17 gene products), *Bradyrhizobium japonicum *(17 gene products), *Mycobacterium tuberculosis *(13 gene products) and *Streptomyces avermitilis *(13 gene products) (Table 1). For effective pathogenesis, these bacteria deploy an extensive arsenal of biomolecules or virulence factors that would allow them to overcome host defense machinery and appropriate their resources. Studies have highlighted that the peptidoglycan turnover and the release of derivative elicitor molecules such as muropeptides, facilitated by the DD-Peptidases plays significant roles in pathogenesis [56]. Antibiotic compounds form a major component of bacterial defense response against invading pathogenic bacteria and thus, the latter would require mechanisms to neutralise such compounds for successful invasion. The significant representation of the DD-peptidase-like proteins (which are likely to include some β-lactamases) in these genomes suggest that some of them may have been recruited as the components of the invasive machinery deployed to neutralise host defenses and facilitate effective pathogenesis.

**Figure 3:**
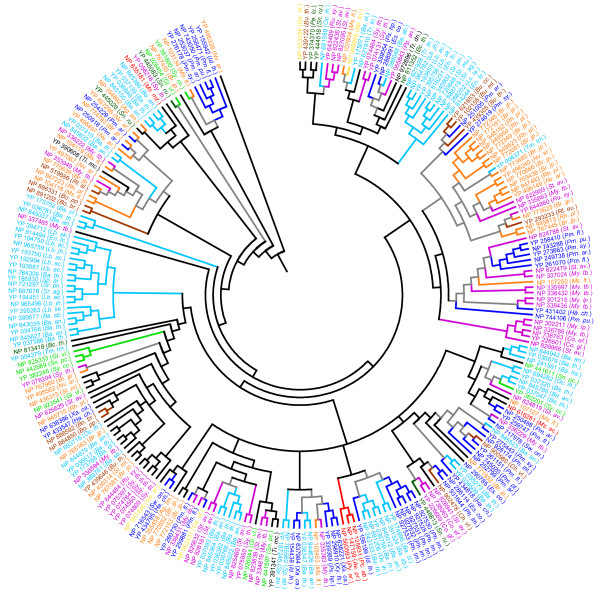
**Phylogenetic analysis of the DD-peptidase-like proteins carried out as described in Figure 1. **The abbreviations and the colour schemes are the same as in Figure 1.

Phylogenetic analysis shows the presence of several clusters of DD-peptidase-like proteins (Figure 3). While several clusters of taxa-specific and species-specific DD-peptidase-like proteins were observed, several clusters were populated by proteins from distinct species. For instance, a DD-peptidase-like protein YP_004371.1 (*Thermus thermophilus HB27*) co-clusters with proteins from Alphaproteobacteria *Bradyrhizobium japonicum *(NP_772081.1, NP_772181.1, NP_772348.1, NP_772349.1, NP_773480.1) and *Rhodopseudomonas palustris *(NP_946954.1, NP_948152, NP_948623.1) suggesting a probable lateral transfer of some DD-peptidase-like genes between these bacterial species. Similarly, NP_822959.1 (*Streptomyces avermitilis*) co-clusters with DD-peptidase-like proteins from Alphaproteobacteria, while YP_156199.1 (*Idiomarina oihiensis*) co-clusters with DD-peptidase-like proteins from *Bacillus sp*. (YP_036759.1, NP_243133.1, NP_845012.1) and *Oceanobacillus iheyensis *(NP_691206.1) (Figure 3). Thus, phylogenetic analysis suggests that the abundance of DD-peptidase-like proteins in prokaryotes was probably facilitated by their dissemination into various prokaryotic species through multiple horizontal gene transfer events.

### Clp protease Family (S14)

Clp proteases are a group of ATP-dependent serine endopeptidases [5]. *E. coli *ClpP is an ATP-dependent serine protease consisting of a smaller protease subunit ClpP, and a larger chaperone regulatory ATPase subunit (either ClpA or ClpX). Though the protease domain is capable of proteolysis on its own, ATPase subunits are essential for effective levels of proteolysis. The catalytic triad residues Ser-His-Asp (S111, H136, D185) are enclosed in a single cavity that allows for degradation of small peptides but precludes the entry of the large folded polypeptides [16,57,58]. Clp proteases do not show any strict specificity for the residues at the P1 or P1' positions in their substrates, but seem to prefer hydrophobic or non-polar residues at these positions [5].

**Figure 4:**
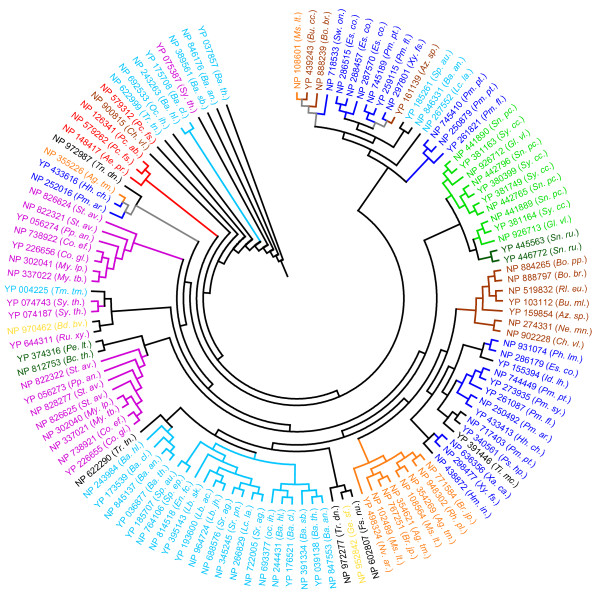
**Phylogenetic analysis of the Clp protease carried out as described in Figure 1**. The abbreviations and the colour schemes are the same as in Figure 1.

A total of 121 Clp protease-like proteins (21 SPHs) were identified in the present study (Tables 1 and 2; Additional file 1). Phylogenetic analysis shows the presence of many clusters of Clp protease-like proteins and significantly populated clusters were identified for Firmicutes, Gammaproteobacteria, Cyanobacteria and Actinobacteria (Figure 4). This is consistent with observations on diversity of Clp protease functions in various prokaryotic lineages [59] that they have been implicated in radioresistance and regulating cell division in *Deinococcus radiodurans *[60], regulating metabolic pathways associated with nutrition in *Bacillus subtilis *[61], regulation of zinc homeostasis in *E. coli *[62], cell viability in cyanobacterium *Synechococcus *[63] and survival during stationary phase in *E. coli *[16]*etc*. Phylogeny also reveals co-clustering of Clp protease-like proteins from distinct species. NP_108601.1 (*Mesorhizobium loti*) co-clusters with YP_439243.1 (*Burkholderia thailandensis*) and NP_888239.1 (*Bordetella bronchiseptica*) suggesting a putative lateral transfer of some Clp protease gene products between these bacterial species. Similarly, YP_161139.1 (*Azoarcus sp EBN1*) co-clusters with NP_297801 (*Xylella fastidiosa*), YP_259115.1 (*Pseudomonas fluorescens*) and NP_745189.1 (*Pseudomonas putida*) suggesting multiple lateral transfer of Clp protease-like proteins between the bacterial species. Yet another instance of probable lateral transfer of Clp proteases was observed with the co-clustering of NP_355226.1 (*Agrobacterium tumefaciens*) with NP_252016.1 (*Pseudomonas aeruginosa*) and NP_433616.1 (*Hahella chejuensis*) (Figure 4) suggesting that the distribution of Clp proteases in prokaryotes may have been facilitated by multiple lateral gene transfer events.

### Lon protease Family (S16)

Lon proteases are a group of ATP-dependent serine proteases, where unlike the Clp proteases, the catalytic protease domain and the ATPase domain reside in the same polypeptide. *E. coli *Lon protease was the first ATP-dependent protease to be described and consists of three functional domains: the N-terminal domain (LON), a central ATPase domain (AAA+ module) and a C-terminal proteolytic domain (Lon_C). The N-terminal LON domain, along with the AAA module, is believed to impart substrate specificity to Lon proteases. Lon proteases employ a Ser-Lys (S679, K/R722) catalytic dyad. Based on the conservation of residues around the catalytic serine residue, Lon proteases maybe divided into two subfamilies LonA (PKDGPSA) and LonB ([E/D]GDSA [S/T]) [57,64]. They display broad sequence specificity in degrading polypeptides, with a slight preference for hydrophobic residues at P1 position [5]. In addition to their role in protein quality control by removal of misfolded proteins, Lon proteases are known to regulate a variety of physiological processes such as cell differentiation, sporulation, pathogenicity and stress response in bacteria [65].

**Figure 5:**
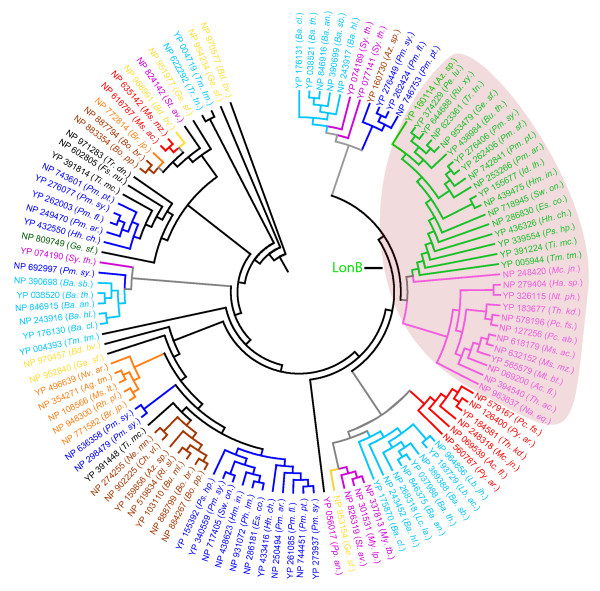
**Phylogenetic analysis of the Lon proteases carried out as described in Figure 1**. Abbreviations and the colour schemes are the same as in Figure 1 except for those employed for phylogenetic clades comprising LonB proteins. Subclusters of bacterial (green) and archaeal (pink) LonB homologues can be visualised.

A total of 117 Lon protease homologues (2 SPHs) were identified in the present analysis. They display a higher representation in Euryarchaeota than trypsins, DD-peptidases and Clp proteases (Tables 1 and 2). Based on the conservation of the residues around the catalytic serine, most Lon protease homologues identified here correspond to the LonA subfamily. However, a significant number of the archaeal and the bacterial Lon proteases were identified as belonging to the LonB subfamily. Phylogenetic analysis reveals several taxa and species-specific clusters of the Lon proteases (Figure 5). Lon protease-like proteins identified as the LonB subfamily members fall into a single cluster, which includes two subclusters of the bacterial and the archaeal Lon proteases. While, most bacterial LonB proteins identified here belong to the Gram-negative bacteria (mostly Gammaproteobacteria), a few homologues were identified in the Gram-positive bacteria (such as YP_644688.1 in *Rubrobacter xylanophilus*) (Figure 5; Additional file 1). Distinct subclusters of archaeal and bacterial LonB members suggest diversification of the LonB repertoire in the two kingdoms as a consequence of the organisms' adaptation to their specific environments. However, phylogeny also reveals co-clustering of bacterial LonB members from distinct species (Figure 5). YP_644688.1 was observed to co-cluster with YP_374229.1 (*Pelodictyon luteolum*; Chlorobi); NP_623361.1 (*Thermoanaerobacter tengcongensis*; Firmicutes); NP_953479.1 (*Geobacter sulfurreducens*; Deltaproteobacteria) *etc *suggesting that some of the LonB-like proteins in bacteria were disseminated to different species through multiple lateral gene transfer events (Figure 5). Similar inferences can be drawn based on the clustering observed for archaeal Lon protease-like proteins. For instance, an archaeal LonB-like protein YP_183677.1 from *Thermococcus kodakaraensis KOD1 *closely associates with NP_127256.1 (*Pyrococcus abyssi*) and NP_578196.1 (*Pyrococcus furiosus*). Similarly, a *Thermococcus kodakaraensis KOD1 *Lon protease-like protein YP_184581.1 co-clusters with NP_126400.1 (*Pyrococcus abyssi*) and NP_579167.1 (*Pyrococcus furiosus*) (Figure 5). Literature reports have suggested the possibility of horizontal gene transfer between *Thermococcus kodakaraensis *and *Pyrococcus sp*. [66] suggesting a lateral transfer of Lon protease-like proteins in the three archaeal species. In another instance, a bacterial Lon protease-like protein NP_968991.1 (*Bdellovibrio bacteriovorus*) co-clusters with two archaeal Lon protease-like proteins NP_616787.1 (*Methanosarcina acetovirans*) and NP_635142.1 (*Methanosarcina mazei*), suggesting the possibility of the lateral transfer of Lon protease genes between bacteria and archaea (Figure 5).

### Analysis of Domain Architectures

Serine protease-like domains often exist as a part of multi-domain polypeptides. In several enzyme families, changes in the domain alliances are known to modulate the enzyme function, usually by altering the substrate specificity or enzyme efficiency. The co-existing domains may also play a key role in the substrate specificity of these proteins, either by facilitating protein-protein interactions or their specific involvement in pathways [67,68]. Such additional modules may introduce newer and more diverse functions for the serine proteases in the various cellular networks. Therefore, an investigation of various domain combinations in serine protease families would be extremely useful in further understanding of their evolution and the biological functions. The domain architectures of serine protease-like proteins identified in the present study were carefully examined using sensitive sequence and profile search procedures and the known functions of the domains tethered to the serine protease domains were taken into consideration to approximate the putative functional associations for the multi-domain serine protease-like proteins. The propensity of the five families to harbour co-existing domains and the tendency for specific co-existing domains was also analysed. The distribution of the varied domain combinations and the known functions of the co-existing domains associated with the serine protease domain in these proteins were employed to obtain insights into their probable biological function associations.

### Single Domain vs Multi-domain Serine Proteases

**Figure 6:**
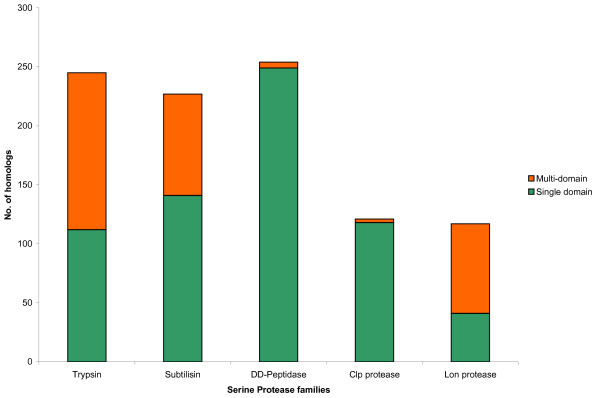
A schematic representation of the abundance of the single domain and the multi-domain serine protease-like proteins in the five serine protease families under study.

Of the 966 serine protease-like proteins identified in the present study, 311 (32%) were found to carry co-existing domains. However, the distribution of the multi-domain proteins is not uniform across the five families, which display unequal preferences to enter into domain alliances. While, trypsins and subtilisins have a significant proportion of the multi-domain representatives, DD-peptidases and Clp proteases are overwhelmingly single-domain polypeptides. Most Lon protease representatives are multi-domain proteins (Figure 6; Table 2). While some protein domain superfamilies are highly versatile and may co-exist with diverse neighbouring domains, some others have a limited repertoire of partner domains [67]. Different domain combinations contribute to functional diversity within and across the lineages [69]. Different propensities of the five serine protease families to form diverse domain combinations may be indicative of selection pressures and possible functional associations. The diversity in the domain combinations and their possible implications for individual serine protease families is discussed below.

### Domain Architectures in the Tryspin (S1) Family

Of the 247 gene products identified as harbouring the trypsin protease-like domains, 133 (54%) were multi-domain polypeptides, where 15 different domain architectures were observed. The impact of some unique domain combinations observed in prokaryotic trypsin-like proteins and their likely influence on the biological functions of these proteins are discussed.

### Trypsins with Protein-Protein Interaction Modules

Analysis of the domain combinations in the trypsin-like proteins identified in the current study reveals the PDZ domains as the most abundant domain partners and their tethering to the C-terminal of the trypsin-like domain. Of the 133 multi-domain trypsin-like proteins identified, 112 were found to have either one (63 of 112) or two (49 of 112) PDZ domains tethered to the C-terminus of the trypsin-like domain (Table 3). PDZ domains are one of the most common protein-protein interaction domains found in diverse organisms from bacteria to humans. They play a major role in the assembly of the multimeric protein complexes involved in cellular signaling and trafficking. This functional role for the PDZ domains is facilitated by their ability to recognise and bind short specific motifs located in the C-termini of the target proteins and/or to the internal peptide sequences, which enables them to recognise and bind to diverse ligands. They modulate the function and the localisation of their associated proteins and are involved in substrate recognition and binding in certain proteases [70-72]. S1-PDZ couple was the only domain combination identified in the four of six archaeal genomes where trypsin-like proteins were identified, suggesting that the additional PDZ domain was recruited later in evolution, possibly in response to the need for bacterial trypsin homologues to be recruited for diverse functions. Other protein interaction modules were also found associated with trypsin-like proteins in prokaryotes. PPC module was found associated with trypsin homologues (YP_434226.1, YP_437990.1 respectively) in *Hahella chejuensis *(Table 3; Additional file 1). PPC is distantly related with PKD module (see below) and is believed to mediate protein-protein and protein-carbohydrate interactions in secreted proteins [5,73]. Four FG-GAP modules, important for ligand binding in certain proteins [74], were found tethered to the C-terminus of the trypsin protease domain in NP_825221.1 in *Streptomyces avermitilis*. ANF-receptor module corresponding to the ligand binding region of several receptors [75] was found associated with a trypsin homologue (YP_073997.1) in *Symbiobacterium thermophilum *[5] (Table 3; Additional file 1).

### Trypsins with Modules Associated with Pathogenesis and Cell Recognition

Many trypsin homologues identified in the present analysis reveal their association with modules that function in cellular recognition and pathogenesis (Table 3; Additional file 1) clearly suggesting the biological role of such trypsin domains in host pathogenesis. For instance, eight trypsin homologues were found associated with Colicin_V domain, N-terminal to the protease domain (Table 3; Additional file 1). Colicin_V domain kills target cells by disrupting their membrane potential [76] and may assist pathogenesis and/or defense. Interestingly, all eight Colicin_V domain containing trypsin-like proteins identified in the present study were found in Gram-positive bacteria of actinobacteria lineage (Table 1; Additional file 1). Perhaps, such domain combinations are required for bacteria that live in harsh conditions since several of them are soil bacteria and may have disseminated to these species via multiple horizontal gene transfer events. NP_344916.1, carrying CW_binding_1 repeat, was identified in *Streptococcus pneumoniae*. This repeat is believed to be important in mediating recognition of choline-containing cell walls [77] (Table 3; Additional file 1).

### Trypsins with modules associated with signalling and metabolism

Some trypsin homologues were found associated with regions most similar to modules likely to function in signalling and metabolism (Table 3; Additional file 1). Trypsin homologue YP_434226.1 (see above) was also found associated with an SCP domain (C-terminal to trypsin and PPC domains) likely to have a calcium chelating function and is involved in many signalling processes [73]. NP_811686.1 from *Bacteroides thetaiotaomicron *is associated with two FHA (forkhead-associated) domains N-terminal to the protease domain, FHA domain which is found in diverse proteins associated with metabolic processes such as DNA repair, signalling, transport *etc *[78]. NP_822175.1 in *Streptomyces avermitilis *was associated with a CBM_5_12 module C-terminal to the protease domain; these are presumed to have a carbohydrate-binding function. YP_273108.1 in *Pseudomonas syringae *was associated with TerD module N-terminal to the protease domain; this domain, found in tellurite resistance proteins, is required for growth in toxic medium. This is not functionally characterised to our knowledge. NP_604177.1 in *Fusobacterium nucleatum *carries an Endonuclease_NS domain, which encodes an endonuclease that acts on double and single-stranded nucleic acids [5] (Table 3; Additional file 1).

### Co-Existing Domains that likely Modulate the Trypsin Protease Domain

Trypsin homologues (NP_822175.1 and NP_827729.1) from *Streptomyces avermitilis *were associated with Alpha lytic protease prodomain (Pro_Al_prot), usually associated with Alpha-lytic endopeptidases – a subset of trypsins involved in lysing and degrading soil organisms (Table 3; Additional file 1). It is required for the correct folding of the adjacent protease domain and acts as an inhibitor of the mature enzyme when attached to the protease domain [79].

### Other Domains associated with the Trypsin Homologues in Prokaryotes

Modules of indeterminate function were also found associated with some trypsin-like proteins. For example YP_374752.1 from *Pelodictyon luteolum *was found associated with six Sel1 repeats that were originally identified in a negative regulator of Notch signalling pathway in *Caenorahbditis elegans *[80]. However, their functions in mammalian species are unknown and the absence of the components of the Notch signalling pathway in prokaryotes suggests their involvement in some other physiological processes.

### Domain Architectures in the Subtilisin (S8) Family

Subtilisins were found to be the most versatile of the five serine protease families. 85 out of 227 (37%) proteins with subtilisin-like domains were identified as multi-domain polypeptides and a total of 38 different domain combinations were discerned, many of which are specific to bacteria or unique to certain prokaryotic species (Table 3).

### Subtilisins with Protein-Protein Interaction Modules

Several subtilisins identified in the present study reveal their association with domains that contain regions facilitating protein-protein interactions (Table 3; Additional file 1). Subtilisin-like proteins containing one (nine gene products; such as YP_154554.1 in *Idiomarina loihiensis*) or two (four gene products; such as YP_341139.1 in *Pseudoaltermonas haloplanktis*) PPC domains C-terminal to the protease domain were identified in different prokaryotic lineages. While the single PPC-domain containing subtilisin-like proteins were identified only in archaea and Gram-negative bacteria (except NP_051605.1 in *Deinococcus radiodurans*, which represents an intermediate between Gram-positive and Gram-negative bacteria), subtilisin-like proteins with two PPC domains were identified in both Gram-positive and Gram-negative bacteria as well as archaea (Tables 1, 3; Additional file 1). Twelve gene products (such as NP_391688.1 in *Bacillus subtilis*) were identified that carry PA domain inserts in the subtilisin protease domain. PA domain is suggested to form a lid-like structure that covers the active site in the protease and is believed to be involved in protein interactions or mediate substrate recognition by proteases [81]. Subtilisin-like proteins (such as YP_326498.1 in *Natromonas pharaonis*) associated with PKD domain were also identified. PKD domains are predicted to be involved in protein-protein and protein-carbohydrate interactions [82] (Table 3; Additional file 1).

### Subtilisins with Modules associated with Pathogenesis and Cell Recognition

Several subtilisin-like proteins identified here were found in association with modules that function in cellular recognition and pathogenesis (Table 3; Additional file 1). For instance, 15 subtilisin-like proteins (such as YP_260308.1 in *Pseudomonas fluorescens*) were identifiedthat carry an Autotransporter beta-domain, C-terminal to the subtilisin domain (Table 3; Additional file 1). This module encodes for a β-barrel domain that usually occurs at the C-terminus of the various domains which it translocates across the outer membrane of the Gram-negative bacteria, sometimes followed by an autocatalytic cleavage of the passenger domain. They are often associated with virulence functions such as cell adhesion and invasion [83]. Interestingly, a subtilisin-like protein associated with an autotransporter module NP_602747.1 was identified in a Gram-positive bacterium *Fusobacterium nucleatum *(Tables 1, 3; Additional file 1). Subtilisin-like proteins with Gram_pos_anchor modules, which helps to gain access to host cells were identified (NP_241562.1 in *Bacillus halodurans*). NP_689039.1 in *Sterptococcus agalacticae *additionally carries a closely related motif called YSIRK type signal peptide [5]. Dockerin I type repeats, which are critical components of cellulosome, that degrades crystalline cellulose [84], were found associated with the subtilisin domain in NP_280653.1 from *Halobacterium*. Cleaved_Adhesin domain found in hemagglutinins and peptidases that in *Porphyromonas *form components of extracellular virulence complex RgpA-Kgp [85] was associated with a subtilisin-like protein YP_074547.1 in *Symbiobacterium thermophilum*. Big_2 domain possibly associated with cell adhesion in bacteria was found encoded by subtilisin-like protein NP_969490.1 in the predatory bacterium *Bdellovibrio bacteriovorus *where it is likely to be associated with the hydrolytic machinery that facilitates the bacterium's predatory lifecycle [40] and in NP_624131.1 in *Thermoanaerobacter tengcongenesis*, which also carries a pair of SLH domains believed to anchor the peptidoglycans [86]. Some other domains associated with cell adhesion were also identified in some subtilisin-like proteins such as HemolysinCabind, which is probably involved in calcium mediated binding to the specific receptors and in the folding of the protein subsequent to the transmemembrane translocation [87] (NP_747027.1 in *Pseudomonas putida*; NP_927988.1 in *Photorhabdus luminescens*); CARDB-cell adhesion related bacterial domain (NP_954260.1 in *Geobacter sulfurreducens*) [5]; Fibronectin type III (fn3) domain involved in cell surface binding [88] (YP_446403.1 in *Salinibacter ruber*) (Table 3; Additional file 1).

### Subtilisins with Modules associated with Signalling and Metabolism

Many subtilisin-like proteins were identified with the regions most similar to the modules likely to function in signalling and metabolism flanking the protease domain (Table 3; Additional file 1). NP_616940.1 gene product in *Methanosarcina acetovirans *(archaea) carries a NosD module C-terminal to the subtilisin domain; NosD is a periplasmic protein believed to insert copper into exported reductase apoenzyme [89]. NP_965819.1, a gene product in *Lactobacillus johnsonii*, encodes a multi-domain protein with five FIVAR modules, a putative sugar binding domain mostly found in cell-wall associated proteins [5]. NP_967057.1 in *Bdellovibrio bacteriovorus *was found to carry a CUB domain, an extracellular module associated with diverse functions in development and signalling in eukaryotes, however, its role in prokaryotes is not clear [90] (Table 3; Additional file 1).

### Co-existing Domains that likely Modulate the Subtilisin Domain

Some subtilisin-like proteins were found associated with the domains that likely modulate the function of the adjacent subtlisin protease domain (Table 3; Additional file 1). A subtilisin-coexisting domain, that occurs N-terminal to many subtilisins including those in plants [23] and is subsequently cleaved prior to activation, was found in several subtilisin-like proteins identified in the present study (such as NP_241550.1 in *Bacillus halodurans*). NP_967370.1 gene product in *Bdellovibrio bacteriovorus *codes for a Proprotein convertase P-domain C-terminal to the subtilisin domain. It is associated with the kex2/subtilisin endopeptidases in eukaryotes, gammaproteobacteria and few others and is believed to be necessary for the folding and maintenance of the subtilisin domain and regulating its calcium pH specificity [91]. NP_394205.1 in *Thermoplasma acidophilum *(archaea) encodes for a thermopsin module, N-terminal to the subtilisin domain, similar to those found in the thermostable acid proteases in archaebacteria [5] (Table 3; Additional file 1).

### Other Domains associated with the Subtilisin Homologues in Prokaryotes

Several modules of unknown or indeterminate function were also found associated with subtilisin-like proteins in prokaryotes (Table 3; Additional file 1). These include GRP module (similar to those in stress-upregulated glycine-rich proteins) in NP_435320.1 in *Sinirhizobium meliloti*; Domain of Unknown Function DUF1034, also associated with some plant subtilisins [23] seen in YP_194362.1 in *Lactobacillus acidophilus*; DUF11 (believed to be involved in porin formation) in NP_951948.1 in *Geobacter sulfurreducens*; BNR repeats in NP_824495.1 in *Streptomyces avermitilis*; PilZ domain in NP_969350.1 in *Bdellovibrio bacteriovorus*; Tryptophan rich (W_rich_C) domain in YP_382882.1 in *Synechococcus *[5] and so on (Table 3; Additional file 1).

### Domain Architectures in DD-peptidase (S12) Family

DD-peptidase-like proteins were found to be extremely rigid in terms of domain combinations. Only five of 254 proteins carrying DD-peptidase-like domains were identified as multi-domain polypeptides, in sharp contrast to other serine protease families analysed here, except Clp proteases (Table 3; Additional file 1). These exceptional prokaryotic DD-peptidase multi-domain architectures are discussed here. DD-peptidase-like protein YP_434618.1 in *Hahella chejuensis *was found to encode a region most similar to ABC transporters that function in translocation of diverse compounds across biological membranes [92]. Another homologue NP_824819.1 in *Streptomyces avermitilis *carries three each of Condensation (associated with enzymes that synthesise peptide antibiotics [93]), AMP-binding (associated with enzymes that act via ATP-dependent AMP binding) and PP-binding (prosthetic group of acyl carrier proteins) modules N-terminal to the predicted DD-peptidase domain. Two DD-peptidase-like proteins identified in the Gram-negative bacteria (Chlorobi), NP_811352.1 (*Bacteroides thetaiotaomicron*) and YP_444518.1 (*Salinibacter ruber*) were found associated with the Glyco_hydro_3 module found in the O-Glycosyl hydrolases that hydrolyse the glycosidic bond between two or more carbohydrates. YP_444518.1 also carries a Glyco_hydro_3_C module, often found in association with Glyco_hydro_3 and is involved in catalysis and binding β-glucan [5,94] (Table 3; Additional file 1).

### Domain Architectures in Clp Protease (S14) Family

Clp proteases show an overwhelming preference for existence as single domain polypeptides. Only three of 121 Clp protease homologues identified in the current study were found to carry additional domains (Table 3; Additional file 1). All three multi-domain Clp homologues NP_148417.1 (*Aeropyrum pernix*), NP_126341.1 (*Pyrococcus abyssi*) and NP_579262.1 (*Pyrococcus furiosus*) were identified in hyperthermophilic archaea and are associated with NfeD-like module C-terminal to the protease domain (Table 3; Additional file 1). NfeD-like domain corresponds to a family of proteins that include nodulation efficiency proteins and protease homologues. Although exact function of this family remains unknown, it is unlikely to be involved specifically in nodulation [5] (Table 3; Additional file 1). The lack of multi-domain polypeptides amongst Clp protease homologues can be viewed in terms of their known functional associations. Clp proteases are known to extensively form complexes with AAA+ (ATPases Associated with diverse cellular Activities) modules, which are one of the most diverse and promiscuous modules known to associate with diverse domains and function in a wide range of physiological processes [95,96]. By extension, the association of Clp protease domains with AAA+ mediated assemblies of protein complexes would allow them to modulate a host of cellular and physiological processes where AAA+ modules are required and would facilitate the availability of diverse substrates for degradation by Clp protease domain. Therefore, it would seem that Clp proteases may rely on forming complexes with AAA+ modules to ensure their association with diverse processes.

### Domain Architectures in Lon Protease (S16) Family

Lon proteases display a marked preference for existence as multi-domain polypeptides. Of the 117 Lon protease-like proteins identified here, 78 (67%) gene products were found to retain co-existing domains and display a conserved domain architecture (Table 3; Additional file 1). Fifty eight of the 117 Lon protease-like proteins were found to possess the canonical Lon protease domain combination, consisting of an N-terminal domain (LON), a central ATPase domain (AAA+ module) and a C-terminal proteolytic domain (Lon_C). The N-terminal LON domain, together with the AAA (ATPase) module, selectively interacts with the target protein and is believed to impart substrate specificity to the Lon proteases [64]. A significant repertoire was found associated with the Sigma54_activat module that has ATPase activity and interacts with the sigma-54 factor involved in the bacterial RNA polymerase mediated transcription initiation [97]. It is likely that the adjacent Lon protease domain may be involved in processing the Sigma54_activat domain. Two predominant architectures were observed for the Sigma54_activat associated Lon protease homologues: Sigma54_activat-S16 architectures found only in the archaeal proteins such as NP_127256.1 (*Pyrococcus abyssi*) and the Sigma54_activat-AAA-S16 in gene products such as YP_183677.1 (*Thermococcus kodakaraensis*) (Table 3; Additional file 1). The Magnesium chelatase, subunit ChlI domain, which is involved in synthesis of the bacteriochlorophyll [98] was found associated with five archaeal Lon protease-like proteins, where it occurs N-terminal to the Lon protease domain (such as NP_248420.1 (*Methanococcus jannaschii*). An archaeal Lon protease-like protein NP_578196.1 (*Pyrococcus furiosus*) carries a Magnesium chelatase and a Sigma54_activat domain N-terminal to the predicted Lon protease domain (Table 3; Additional file 1). Interestingly, all the Mg_chelatase domain containing Lon protease-like proteins are assigned as the putative members of the LonB subfamily of Lon proteases based on the sequence and phylogenetic analysis (Figure 5; Additional file 1), suggesting a specific acquisition of the Mg_chelatase module by some archaeal proteases belonging the LonB subfamily. A Lon protease-like protein YP_160730.1 from *Azoarcus *was found associated with the DnaB helicase C-terminal domain that unwinds the DNA duplex in the prokaryotes. The domain contains an ATP-binding site and is a likely site for ATP hydrolysis [5]. Its co-occurrence with the Lon protease domain suggests a putative alternate mechanism for facilitating ATP-dependent Lon protease activity. NP_389388.1 in *Bacillus subtilis *was found associated with a PDZ domain (see above) N-terminal to Lon protease domain, which may facilitate interactions with specific substrates (Table 3; Additional file 1).

## Conclusion

Genome-wide studies reveal a large number of serine proteases belonging to the trypsin, subtilisin, DD-peptidase, Clp protease and Lon protease families in prokaryotes. However, there is only a limited knowledge available about their probable biological functions. Trypsins, subtilisins and the DD-peptidases have a higher number of representatives than the Clp protease and the Lon protease families in the genomes considered for the present analysis. The differences in the representations of the five serine protease families probably arose due to the selection of specific classes of serine proteases during evolution as an adaptation to different cellular and extracellular environments. For instance, the high abundance of the trypsins and the subtilisins in *Bdellovibrio bacteriovorus *is likely due to their involvement as the components of the hydrolytic arsenal deployed for pathogenesis by the bacterium. Similarly, the abundance of the DD-peptidase-like proteins in some pathogenic bacteria (such as *Streptomyces avermitilis*) suggests their probable functions as virulence factors and in antibiotic resistance. Interestingly, while trypsins are also well represented in the eukaryotes, subtilisins (with the exception of plants) and DD-peptidases are less abundant in higher organisms suggesting that such enzymes were likely lost during the evolution as an adaptation to the cellular (and the extracellular) environment in the eukaryotes. Phylogenetic analysis suggests putative lateral transfer of serine protease genes between different bacterial and archaeal species and also between some bacteria and archaea. It is likely that some serine protease-like proteins may have been disseminated in the different prokaryotic species through probable horizontal gene transfer events. The lateral transfer of the serine protease genes in bacteria may possibly confer an evolutionary advantage on the recipient [99].

In the absence of the experimental characterisation for the most of the proteins sequences, an approximation of their biological functions is often inferred based on their sequence similarities to the proteins of known function. Studies have shown that the overall biological functions and the interactions of the multi-domain proteins are conserved by the retention of the domain composition and sequential arrangement [100]. Therefore the domain architectures of the multi-domain serine protease-like proteins were investigated to obtain insights into their probable functional associations. A differential distribution of the multi-domain proteins across the five families indicates different selection pressures and possible functional associations. Enzymatic and non-enzymatic domains such as those associated with protein interaction, signaling, pathogenesis, cell adhesion, metabolism *etc *were found tethered to the serine protease domain. Addition of new domains would permit these enzymes to acquire new functions and specificities contributing to the functional diversities of these gene families. However, a lack of significant repertoire of accessory domains does not necessarily indicate lack of functional diversity. Enzyme families may adopt alternative mechanisms to expand their functional repertoire, such as associating with limited but functionally diverse modules and other proteins or effecting changes in key amino acid residues. For instance, the Clp proteases form extensive complexes with the functionally diverse AAA+ modules that would enable them to modulate various physiological processes. The presence of multiple copies of the same accessory domain (that probably arose due to internal tandem duplication or equivalent events) in many serine protease-like proteins is another likely approach to expand their functional repertoire. Some domain combinations (such as S1-PDZ; LON-AAA-S16 *etc*.) were found to be widespread and conserved in prokaryotes suggesting a critical roles. Unique domain combinations of some prokaryotic serine protease-like proteins suggest their involvement in species-specific functions. Several domain architectures identified in prokaryotic serine proteases in the present analysis are very different from those reported in eukaryotic serine proteases. This highlights the distinct biological roles for the prokaryotic serine proteases compared to those in the eukaryotes. Some of these prokaryotic serine protease-like proteins with atypical domain combinations are attractive targets for experimental characterisation. Some pathogen peptidases identified in the present analysis with no identifiable homologues (unique domain architectures) in their hosts may be promising drug targets [99]. For example, a putative trypsin NP_344916.1 (Tryp-CW_binding-CW_binding) and a subtilisin-like protein NP_345151.1 (Sub_N-Subt-PA-DUF1034-Gram_pos_anchor) in *Streptococcus pneumoniae*, a human pathogen, are postulated to function in pathogenesis based on the domains associated with the serine protease domain. Some serine protease-like proteins such as NP_967057.1 (CUB domain) in *Bdellovibrio bacteriovorus *and YP_374752.1 in *Pelodictyon luteolum *(Sel1 repeats) are associated with domains similar to eukaryotic signalling modules with no known functions in prokaryotes. A systematic deletion of the one or more co-existing domains in the gene products with atypical domain combinations and the resulting phenotypes may help understand their roles in pathogenesis and other prokaryotic physiological processes and the role of the co-existing domains in modulating the functions of these serine proteases. Similarly, a phylogenetic cluster of the trypsin-like proteins (such as NP_302493.1 in *Mycobacterium leprae*) that contain a Colicin_V domain known to function in pathogenesis, tethered C-terminal to the protease domain, suggests an acquisition of the unique patterns in the interface region of the trypsin domain in these gene products. Identification of the conserved domain-domain interface regions and mutagenesis may help understand the function of these gene products and the role of the interactions between the adjacent domains.

The systematic analysis of the five serine protease families in the representative prokaryotic genomes is expected to enable a better understanding of the previously uncharacterised serine proteases encoded in the various genomes. The numbers of the serine protease-like proteins is likely to increase with the increasing amounts of the prokaryotic genomic data and the present analysis should help provide paradigms that would be useful in extending such analyses to a broader repertoire of the prokaryotes. The diversity of the functional domains co-existing with the protease domain in the serine protease-like proteins has provided clues to their biological functions, much of which are yet to be characterised experimentally. Experimental characterisation of some of these gene products as proposed here may help uncover the specific functional roles for the serine proteases in various cellular and physiological processes and help understand their influence on growth and development in the prokaryotic species.

## Abbreviations

ATP: Adenosine triphosphate; NCBI: National Center for Biotechnology Information

## Authors' contributions

LT and RS conceived of the study and participated in its design and coordination. LT carried out the computational sequence analysis. LT authored the first draft of this manuscript and RS provided comments and revisions to the final version of this text. Both authors read and approved the final manuscript.

## Supplementary Material

Additional file 1 **An inventory of the serine protease-like proteins belonging to the five chosen families identified in the various prokaryotic genomes and their inferred domain architectures**. An inventory of serine proteases belonging to five chosen families identified in various prokaryotic genomes using the multi-fold approach (see Methods for details) and the details of co-existing domains (adjacent to the serine protease domain) identified for each putative serine protease. Click here for file

Additional file 2 **A list of the various prokaryotic genomes considered for the present study**. The species abbreviations employed in the illustrations of the phylogenetic trees accompany the species name in parentheses. A list of the genomes in which serine protease-like proteins were identified in the current study. The genomes have been categorized accoding to their taxonomic lineages and the abbreviated species IDs (in parenthesis) that have been suffixed to the protein identifiers in the phylogenetic trees accompany the species name. Click here for file
