# Supplementary material for: Genome-wide survey of prokaryotic serine proteases: Analysis of distribution and domain architectures of five serine protease families in prokaryotes
Source: BMC Genomics. 2008 Nov 19;9:549. doi: 10.1186/1471-2164-9-549 (PMC2605481; doi:10.1186/1471-2164-9-549)
Supplement: Additional file 1 — An inventory of the serine protease-like proteins belonging to the five chosen families identified in the various prokaryotic genomes and their inferred domain architectures. An inventory of serine proteases belonging to five chosen families identified in various prokaryotic genomes using the multi-fold approach (see Methods for details) and the details of co-existing domains (adjacent to the serine protease domain) identified for each putative serine protease. [file 1471-2164-9-549-S1.doc]

Additional file S1: An inventory of the serine protease-like proteins belonging to the five chosen families identified in the various prokaryotic genomes and their inferred domain architectures

| Prokaryotic Genomes | Domain Architecture^#^ |
| --- | --- |
| Aeropyrum_pernix |  |
| Subtilisin family |  |
| NP_147093.1 | Subt |
| NP_147357.1 | Subt |
| NP_147793.1 | Subt |
|  |  |
| DD-Peptidase family |  |
| NP_147159.1 | DDPept |
|  |  |
| Clp protease family |  |
| NP_148417.1 | Clp-NfeD |
|  |  |
| Agrobacterium_tumefaciens_C58_Cereon |  |
| Trypsin family |  |
| NP_354001.1 | Tryp-PDZ-PDZ |
| NP_354890.1 | Tryp-PDZ-PDZ |
| NP_355011.1 | Tryp-PDZ-PDZ |
|  |  |
| DD-Peptidase family |  |
| NP_353949.1 (**SPH; Y190D**) | DDPept |
| NP_354892.1 | DDPept |
| NP_355459.1 | DDPept |
|  |  |
| Clp protease family |  |
| NP_354269.1 | Clp |
| NP_354621.1 | Clp |
| NP_355226.1 | Clp |
|  |  |
| Lon protease family |  |
| NP_354271.1 | LON-AAA-Lon_C |
|  |  |
| Archaeoglobus_fulgidus |  |
| Subtilisin family |  |
| NP_070480.1 (**SPH; D137-**) | Subt-PKD-PKD |
| NP_070481.1 (**SPH; S325-**) | Sub_N-Subt |
|  |  |
| Lon protease family |  |
| NP_069200.1 (Lon_B) | Sigma54_activat-Lon_C |
| NP_069539.1 | Lon_C |
|  |  |
| Azoarcus_sp_EbN1 |  |
| Trypsin family |  |
| YP_157036.1 | Tryp |
| YP_157710.1 | Tryp-PDZ |
| YP_158697.1 | Tryp-PDZ-PDZ |
| YP_160140.1 | Tryp-PDZ-PDZ |
|  |  |
| Subtilisin family |  |
| YP_157323.1 | Subt |
| YP_157673.1 | Subt |
| YP_159971.1 | Subt |
|  |  |
| Clp protease family |  |
| YP_159854.1 | Clp |
| YP_161139.1 | Clp |
|  |  |
| Lon protease family |  |
| YP_159856.1 | LON-AAA-Lon_C |
| YP_160114.1 (Lon_B) | Lon_C |
| YP_160730.1 (**SPH; S679A**) | DnaB-Lon_C |
|  |  |
| Bacillus_anthracis_Ames |  |
| Trypsin family |  |
| NP_845925.1 | Tryp |
| NP_847856.1 | Tryp |
|  |  |
| Subtilisin family |  |
| NP_844405.1 | Subt |
| NP_844762.1 | Sub_N-Subt |
| NP_846139.1 | PPC-Subt |
| NP_846805.1 | Sub_N-Subt-PA |
|  |  |
| DD-Peptidase family |  |
| NP_843039.1 | DDPept |
| NP_844626.1 | DDPept |
| NP_844879.1 | DDPept |
| NP_844942.1 | DDPept |
| NP_845012.1 | DDPept |
| NP_845022.1 | DDPept |
| NP_845037.1 | DDPept |
| NP_845100.1 | DDPept |
| NP_845364.1 | DDPept |
| NP_845386.1 | DDPept |
| NP_845523.1 | DDPept |
| NP_845632.1 | DDPept |
| NP_845786.1 | DDPept |
| NP_845827.1 | DDPept |
|  |  |
| Clp protease family |  |
| NP_845137.1 | Clp |
| NP_846176.1 | Clp |
| NP_846331.1 (**SPH; D185E**) | Clp |
| NP_847553.1 | Clp |
|  |  |
| Lon protease family |  |
| NP_846375.1 | Lon_C |
| NP_846915.1 | LON-AAA-Lon_C |
| NP_846916.1 | Sigma54_activat-AAA-Lon_C |
|  |  |
| Bacillus_clausii_KSM-K16 |  |
| Trypsin family |  |
| YP_175598.1 | Tryp |
| YP_177585.1 | Tryp-PDZ |
|  |  |
| Subtilisin family |  |
| YP_174252.1 | Sub_N-Subt-PA |
| YP_174261.1 | Sub_N-Subt |
| YP_175254.1 | Subt |
| YP_176948.1 | Subt |
| YP_177352.1 | Subt-PA |
|  |  |
| DD-Peptidase family |  |
| YP_173752.1 | DDPept |
| YP_173768.1 | DDPept |
| YP_174895.1 | DDPept |
| YP_175071.1 | DDPept |
|  |  |
| Clp protease family |  |
| YP_173539.1 | Clp |
| YP_175708.1 | Clp |
| YP_176521.1 | Clp |
|  |  |
| Lon protease family |  |
| YP_175870.1 | PDZ-Lon_C |
| YP_176130.1 | LON-AAA-Lon_C |
| YP_176131.1 | AAA-Lon_C |
|  |  |
| Bacillus_halodurans |  |
| Trypsin family |  |
| NP_243055.1 | Tryp |
| NP_244890.1 | Tryp |
|  |  |
| Subtilisin family |  |
| NP_241550.1 | Sub_N-Subt |
| NP_241562.1 | Subt-Gram_pos_anchor |
| NP_241697.1 | Sub_N-Subt-PA |
| NP_241721.1 | Sub_N-Subt |
| NP_242357.1 | Subt |
| NP_242796.1 | Subt |
| NP_242946.1 | Subt |
| NP_243049.1 | Subt |
| NP_244630.1 | Subt-PA |
|  |  |
| DD-Peptidase family |  |
| NP_241581.1 | DDPept |
| NP_243133.1 | DDPept |
|  |  |
| Clp protease family |  |
| NP_243263.1 | Clp |
| NP_243984.1 | Clp |
| NP_244431.1 | Clp |
|  |  |
| Lon protease family |  |
| NP_243452.1 | PDZ-Lon_C |
| NP_243916.1 | LON-AAA-Lon_C |
| NP_243917.1 | Sigma54_activat-AAA-Lon_C |
|  |  |
| Bacillus_subtilis |  |
| Trypsin family |  |
| NP_388106.1 | Tryp |
| NP_389173.1 | Tryp |
| NP_391180.2 | Tryp-PDZ |
| NP_391916.1 | Tryp |
|  |  |
| Subtilisin family |  |
| NP_388911.1 | Sub_N-Subt |
| NP_388958.1 | Subt |
| NP_389202.1 | Subt |
| NP_389413.1 | Sub_N-Subt |
| NP_389608.1 | Subt |
| NP_391688.1 | Sub_N-Subt-PA |
| NP_391719.1 | Sub_N-Subt |
|  |  |
| DD-Peptidase family |  |
| NP_388048.1 | DDPept |
| NP_389577.1 | DDPept |
| NP_389761.1 | DDPept |
| NP_391324.1 | DDPept |
|  |  |
| Clp protease family |  |
| NP_389561.2 | Clp |
| NP_391334.1 | Clp |
|  |  |
| Lon protease family |  |
| NP_389388.1 | PDZ-Lon_C |
| NP_390698.1 | LON-AAA-Lon_C |
| NP_390699.1 | LON-AAA-Lon_C |
|  |  |
| Bacillus_thuringiensis_konkukian |  |
| Trypsin family |  |
| YP_037679.1 | Tryp |
| YP_039451.1 | Tryp |
|  |  |
| Subtilisin family |  |
| YP_035204.1 | Subt |
| YP_036161.1 | Subt |
| YP_036482.1 | Sub_N-Subt |
| YP_037822.1 | PPC-Subt |
| YP_038408.1 | Sub_N-Subt-PA |
|  |  |
| DD-Peptidase family |  |
| YP_034509.1 | DDPept |
| YP_034766.1 | DDPept |
| YP_035273.1 | DDPept |
| YP_036347.1 | DDPept |
| YP_036382.1 | DDPept |
| YP_036617.1 | DDPept |
| YP_036679.1 | DDPept |
| YP_036759.1 | DDPept |
| YP_036766.1 | DDPept |
| YP_036781.1 | DDPept |
| YP_036846.1 | DDPept |
| YP_037093.1 | DDPept |
| YP_037139.1 | DDPept |
| YP_037320.1 | DDPept |
| YP_037410.1 | DDPept |
| YP_037547.1 | DDPept |
| YP_037586.1 | DDPept |
|  |  |
| Clp protease family |  |
| YP_036877.1 | Clp |
| YP_037857.1 | Clp |
| YP_039138.1 | Clp |
|  |  |
| Lon protease family |  |
| YP_037988.1 | Lon_C |
| YP_038520.1 | LON-AAA-Lon_C |
| YP_038521.1 | Sigma54_activat-AAA-Lon_C |
|  |  |
| Bacteroides_thetaiotaomicron_VPI-5482 |  |
| Trypsin family |  |
| NP_809678.1 | Tryp |
| NP_810225.1 | Tryp-PDZ-PDZ |
| NP_811686.1 | FHA-FHA-Tryp |
|  |  |
| Subtilisin family |  |
| NP_809125.1 | Subt-Big_2 |
| NP_812800.1 | Subt |
|  |  |
| DD-Peptidase family |  |
| NP_811352.1 | Glyco_Hydro_3-DDPept |
| NP_813418.1 (**SPH; S93Y**) | DDPept |
|  |  |
| Clp protease family |  |
| NP_812753.1 | Clp |
|  |  |
| Lon protease family |  |
| NP_809749.1 | Lon_C |
|  |  |
| Bdellovibrio_bacteriovorus |  |
| Trypsin family |  |
| NP_967063.1 | Tryp |
| NP_967541.1 | Tryp |
| NP_967599.1 | Tryp |
| NP_967672.1 | Tryp |
| NP_967871.1 | Tryp |
| NP_967935.1 | Tryp |
| NP_967980.1 | Tryp |
| NP_968258.1 | Tryp |
| NP_968289.1 | Tryp |
| NP_968428.1 | Tryp |
| NP_968757.1 | Tryp |
| NP_968819.1 | Tryp |
| NP_969160.1 | Tryp |
| NP_969162.1 | Tryp |
| NP_969341.1 | Tryp |
| NP_969427.1 | Tryp |
| NP_969430.1 | Tryp |
| NP_969590.1 | Tryp-PDZ-PDZ |
| NP_969708.1 | Tryp |
| NP_970154.1 | Tryp |
| NP_970214.1 | Tryp |
| NP_970215.1 | Tryp |
| NP_970221.1 | Tryp |
|  |  |
| Peptidase_Subt |  |
| NP_967057.1 | Subt-CUB |
| NP_967370.1 | Subt-PProt |
| NP_967505.1 | Subt |
| NP_968187.1 | Subt |
| NP_968328.1 | Subt |
| NP_968340.1 | Subt-PA |
| NP_969106.1 | Subt-PA |
| NP_969150.1 | Subt |
| NP_969249.1 | Subt |
| NP_969350.1 | Subt-PilZ |
| NP_969490.1 | Subt-Big_2 |
| NP_969621.1 | Subt |
| NP_969857.1 | Subt |
| NP_969995.1 | Subt-PA |
| NP_970560.1 |  |
|  |  |
| DD-Peptidase family |  |
| NP_967426.1 | DDPept |
| NP_968808.1 | DDPept |
|  |  |
| Clp protease family |  |
| NP_970462.1 | Clp |
|  |  |
| Lon protease family |  |
| NP_968991.1 | LON-AAA-Lon_C |
| NP_970457.1 | LON-AAA-Lon_C |
| NP_970577.1 | LON-AAA-Lon_C |
|  |  |
| Bordetella_bronchiseptica |  |
| Trypsin family |  |
| NP_890284.1 | Tryp-PDZ-PDZ |
| NP_891400.1 | Tryp-PDZ |
|  |  |
| Subtilisin family |  |
| NP_886968.1 | Subt-Autotrans |
| NP_887460.1 | Subt |
|  |  |
| DD-Peptidase family |  |
| NP_888592.1 | DDPept |
| NP_891202.1 | DDPept |
|  |  |
| Clp protease family |  |
| NP_888239.1 (**SPH; D185E**) | Clp |
| NP_888797.1 | Clp |
|  |  |
| Lon protease family |  |
| NP_887794.1 | LON-AAA-Lon_C |
| NP_888799.1 | LON-AAA-Lon_C |
|  |  |
| Bordetella_parapertussis |  |
| Trypsin family |  |
| NP_885465.1 | Tryp-PDZ-PDZ |
| NP_886409.1 | Tryp-PDZ |
|  |  |
| Subtilisin family |  |
| NP_882769.1 | Subt-Autotrans |
|  |  |
| DD-Peptidase family |  |
| NP_884830.1 | DDPept |
| NP_886331.1 | DDPept |
|  |  |
| Clp protease family |  |
| NP_884265.1 | Clp |
|  |  |
| Lon protease family |  |
| NP_883354.1 | LON-AAA-Lon_C |
| NP_884267.1 | LON-AAA-Lon_C |
|  |  |
| Bradyrhizobium_japonicum |  |
| Trypsin family |  |
| NP_767489.1 | Tryp |
| NP_767603.1 | Tryp |
| NP_768763.1 | Tryp |
| NP_769231.1 | Tryp-PDZ-PDZ |
| NP_769361.1 | Tryp-PDZ |
| NP_769770.1 | Tryp-PDZ-PDZ |
| NP_771279.1 | Tryp |
| NP_771875.1 | Tryp-PDZ |
| NP_772008.1 | Tryp-PDZ-PDZ |
| NP_773146.1 | Tryp-PDZ-PDZ |
|  |  |
| Subtilisin family |  |
| NP_767334.1 (**SPH; H168D**) | Subt |
| NP_768934.1 | Subt |
| NP_769684.1 | Subt |
|  |  |
| DD-Peptidase family | DDPept |
| NP_767445.1 | DDPept |
| NP_767455.1 | DDPept |
| NP_767581.1 | DDPept |
| NP_767960.1 | DDPept |
| NP_769438.1 | DDPept |
| NP_770552.1 | DDPept |
| NP_770639.1 | DDPept |
| NP_770666.1 | DDPept |
| NP_771932.1 | DDPept |
| NP_772081.1 | DDPept |
| NP_772153.1 | DDPept |
| NP_772181.1 | DDPept |
| NP_772348.1 | DDPept |
| NP_772349.1 | DDPept |
| NP_773480.1 | DDPept |
| NP_773623.1 | DDPept |
| NP_774793.1 | DDPept |
|  |  |
| Clp protease family |  |
| NP_767251.1 | Clp |
| NP_771584.1 | Clp |
|  |  |
| Lon protease family |  |
| NP_771582.1 | LON-AAA-Lon_C |
| NP_772814.1 | LON-AAA-Lon_C |
|  |  |
| Burkholderia_cenocepacia_AU_1054 |  |
| Trypsin family |  |
| YP_620533.1 | Tryp-PDZ-PDZ |
| YP_621880.1 | Tryp-PDZ-PDZ |
| YP_622539.1 | Tryp-PDZ |
|  |  |
| Subtilisin family |  |
| YP_620351.1 | Subt |
|  |  |
| DD-Peptidase family |  |
| YP_621853.1 | DDPept |
|  |  |
| Burkholderia_mallei_ATCC_23344 |  |
| Trypsin family |  |
| YP_102134.1 | Tryp-PDZ-PDZ |
| YP_102335.1 | Tryp-PDZ-PDZ |
| YP_104221.1 | Tryp-PDZ |
|  |  |
| Subtilisin family |  |
| YP_103178.1 | Subt |
| YP_103730.1 | Subt |
|  |  |
| DD-Peptidase family |  |
| YP_102158.1 | DDPept |
|  |  |
| Clp protease family |  |
| YP_103112.1 | Clp |
|  |  |
| Lon protease family |  |
| YP_103110.1 | LON-AAA-Lon_C |
|  |  |
| Burkholderia_thailandensis_E264 |  |
| Subtilisin family |  |
| YP_438579.1 | Subt |
| YP_438599.1 | Subt |
| YP_438845.1 | Subt |
| YP_439008.1 | Subt |
| YP_439613.1 | Subt |
| YP_439625.1 | Subt |
|  |  |
| DD-Peptidase family |  |
| YP_439122.1 | DDPept-DUF1343 |
| YP_439305.1 | DDPept |
| YP_439646.1 | DDPept |
|  |  |
| Clp protease family |  |
| YP_439243.1 (**SPH; D185E**) | Clp |
|  |  |
| Lon protease family |  |
| YP_438984.1 (Lon_B) | Lon_C |
|  |  |
| Chromobacterium_violaceum |  |
| Trypsin family | Tryp |
| NP_901010.1 | Tryp |
| NP_901558.1 | Tryp |
| NP_901731.1 | Tryp |
| NP_902964.1 | Tryp-PDZ-PDZ |
| NP_903516.1 | Tryp |
| NP_903894.1 | Tryp |
|  |  |
| Subtilisin family |  |
| NP_901635.1 | Subt |
| NP_901638.1 | Subt |
| NP_902387.1 | Subt-Pprot |
| NP_903189.1 | Subt |
|  |  |
| DD-Peptidase family |  |
| NP_900980.1 | DDPept |
| NP_902478.1 | DDPept |
|  |  |
| Clp protease family |  |
| NP_900815.1 | Clp |
| NP_902228.1 | Clp |
|  |  |
| Lon protease family |  |
| NP_902225.1 | LON-AAA-Lon_C |
|  |  |
| Corynebacterium_efficiens_YS-314 |  |
| Trypsin family |  |
| NP_736902.1 | Colicin_V-Tryp |
| NP_737418.1 | Tryp |
| NP_737560.1 | Tryp-PDZ |
| NP_737759.1 | Tryp |
| NP_738224.1 | Tryp |
| NP_738541.1 | Tryp-PDZ |
| NP_739220.1 | Tryp |
|  |  |
| Subtilisin family |  |
| NP_737188.1 | Subt |
|  |  |
| DD-Peptidase family |  |
| NP_738063.1 | DDPept |
| NP_738763.1 | DDPept |
|  |  |
| Clp protease family |  |
| NP_738921.1 | Clp |
| NP_738922.1 | Clp |
|  |  |
| Corynebacterium_glutamicum_ATCC_13032_Bielefeld |  |
| Trypsin family |  |
| YP_224595.1 | Colicin_V-Tryp |
| YP_225082.1 | Tryp |
| YP_225167.1 | Tryp-PDZ |
| YP_225385.1 | Tryp |
| YP_225634.1 | Tryp |
| YP_225772.1 | Tryp |
| YP_225998.1 | Tryp |
|  |  |
| Subtilisin family |  |
| YP_224866.1 | Subt |
|  |  |
| DD-Peptidase family |  |
| YP_226501.1 | DDPept |
| YP_226659.1 | DDPept |
|  |  |
| Clp protease family |  |
| YP_226655.1 | Clp |
| YP_226656.1 | Clp |
|  |  |
| Deinococcus_radiodurans |  |
| Subtilisin family |  |
| NP_051605.1 | Subt-PPC |
|  |  |
| Enterococcus_faecalis_V583 |  |
| Trypsin family |  |
| NP_815515.1 | Tryp |
| NP_816640.1 | Tryp-PDZ |
|  |  |
| DD-Peptidase family |  |
| NP_814244.1 | DDPept |
| NP_814494.1 | DDPept |
| NP_816231.1 | DDPept |
|  |  |
| Clp protease family |  |
| NP_814518.1 | Clp |
|  |  |
| Escherichia_coli_O157H7_EDL933 |  |
| Trypsin family |  |
| NP_285857.1 | Tryp-PDZ-PDZ |
| NP_288033.1 | Tryp |
| NP_289802.1 | Tryp-PDZ-PDZ |
| NP_289803.1 | Tryp-PDZ |
|  |  |
| DD-Peptidase family |  |
| NP_286114.1 | DDPept |
| NP_288991.1 | DDPept |
| NP_290785.1 | DDPept |
|  |  |
| Clp protease family |  |
| NP_286179.1 | Clp |
| NP_286515.1 (**SPH; D185E**) | Clp |
| NP_287570.1 (**SPH; D185E**) | Clp |
| NP_288457.1 (**SPH; D185E**) | Clp |
|  |  |
| Lon protease family |  |
| NP_286181.1 | LON-AAA-Lon_C |
| NP_286830.1 (Lon_B) | Lon_C |
|  |  |
| Fusobacterium_nucleatum |  |
| Trypsin family |  |
| NP_604177.1 | Tryp-Endonucl_NS |
|  |  |
| Subtilisin family |  |
| NP_602747.1 | Subt-Autotrans |
| NP_602885.1 | Subt |
|  |  |
| Clp protease family |  |
| NP_602807.1 | Clp |
|  |  |
| Lon protease family |  |
| NP_602805.1 | LON-AAA-Lon_C |
|  |  |
| Geobacter_sulfurreducens |  |
| Trypsin family |  |
| NP_951142.1 | Tryp-PDZ-PDZ |
| NP_951391.1 | Tryp-PDZ-PDZ |
|  |  |
| Subtilisin family |  |
| NP_951948.1 | Subt-PPC |
| NP_953124.1 | Sub_N-Subt |
| NP_954260.1 | Subt-(CARDB)^9^ |
|  |  |
| DD-Peptidase family |  |
| NP_952431.1 | DDPept |
| NP_953096.1 | DDPept |
|  |  |
| Clp protease family |  |
| NP_952842.1 | Clp |
|  |  |
| Lon protease family |  |
| NP_951977.1 | LON-AAA-Lon_C |
| NP_952840.1 | LON-AAA-Lon_C |
| NP_953154.1 (**SPH; S679E; K/R722P**) | Lon_C |
| NP_953479.1 (Lon_B) | Lon_C |
| NP_954234.1 | LON-AAA-Lon_C |
|  |  |
| Gloeobacter_violaceus |  |
| Trypsin family |  |
| NP_924281.1 | Tryp-PDZ |
| NP_924793.1 | Tryp-PDZ |
| NP_925043.1 | Tryp |
| NP_925053.1 | Tryp |
| NP_925645.1 | Tryp |
| NP_926204.1 | Tryp |
| NP_926687.1 | Tryp |
|  |  |
| Subtilisin family |  |
| NP_924001.1 | Subt |
| NP_924223.1 | Subt-(FG-GAP)^5^ |
| NP_924500.1 | Subt |
| NP_926033.1 | Subt-PA |
|  |  |
| DD-Peptidase family |  |
| NP_923541.1 | DDPept |
| NP_925333.1 | DDPept |
| NP_926044.1 | DDPept |
| NP_926454.1 | DDPept |
|  |  |
| Clp protease family |  |
| NP_926712.1 | Clp |
| NP_926713.1 (**SPH; S111G; H136Y; D185L**) | Clp |
|  |  |
| Haemophilus_influenzae |  |
| Trypsin family |  |
| NP_439105.1 | Tryp-PDZ |
| NP_439414.1 | Tryp-PDZ-PDZ |
|  |  |
| Clp protease family |  |
| NP_438872.2 | Clp |
|  |  |
| Lon protease family |  |
| NP_438623.1 | LON-AAA-Lon_C |
| NP_439475.1 (Lon_B) | Lon_C |
|  |  |
| Hahella_chejuensis_KCTC_2396 |  |
| Trypsin family |  |
| YP_432417.1 | Tryp-PDZ-PDZ |
| YP_433063.1 | Tryp-PDZ-PDZ |
| YP_433507.1 | Tryp |
| YP_434226.1 | Tryp-PPC-SCP |
| YP_435307.1 | Tryp-(Sel1)^5^ |
| YP_436401.1 | Tryp-PDZ |
| YP_437957.1 | Tryp |
| YP_437990.1 | Tryp-PPC-PPC |
|  |  |
| Subtilisin family |  |
| YP_432968.1 | Subt-PPC |
| YP_434175.1 | Subt-P_proprotein-P_proprotein |
| YP_435045.1 | Subt |
| YP_436813.1 | PPC-Subt |
|  |  |
| DD-Peptidase family |  |
| YP_431402.1 | DDPept |
| YP_433547.1 | DDPept |
| YP_434618.1 | DDPept-ABC_Trans |
| YP_434795.1 | DDPept |
|  |  |
| Clp protease family |  |
| YP_433413.1 | Clp |
| YP_433616.1 | Clp |
|  |  |
| Lon protease family |  |
| YP_432550.1 | LON-AAA-Lon_C |
| YP_433416.1 | LON-AAA-Lon_C |
| YP_436326.1 (Lon_B) | Lon_C |
|  |  |
| Halobacterium_sp_NRC1 |  |
| Trypsin family |  |
| NP_279350.1 | Tryp |
|  |  |
| Subtilisin family |  |
| NP_280653.1 | Subt-Dock1 |
| NP_281139.1 | Subt-PPC |
|  |  |
| Lon protease family |  |
| NP_279404.1 (Lon_B) | Sigma54_activat-AAA-Lon_C |
|  |  |
| Idiomarina_loihiensis_L2TR |  |
| Trypsin family |  |
| YP_154802.1 | Tryp-PDZ |
| YP_154803.1 | Tryp-PDZ-PDZ |
|  |  |
| Subtilisin family |  |
| YP_154553.1 | Subt-P_proprotein-PKD |
| YP_154554.1 | Subt-PPC |
|  |  |
| DD-Peptidase family |  |
| YP_154438.1 | DDPept |
| YP_155942.1 | DDPept |
| YP_156199.1 | DDPept |
|  |  |
| Clp protease family |  |
| YP_155394.1 | Clp |
|  |  |
| Lon protease family |  |
| YP_155392.1 | LON-AAA-Lon_C |
| YP_155677.1 (Lon_B) | LON-AAA-Lon_C |
|  |  |
| Lactobacillus_acidophilus_NCFM |  |
| Trypsin family |  |
| YP_193016.1 | Tryp-PDZ |
|  |  |
| Subtilisin family |  |
| YP_194362.1 | Subt-DUF1034 |
|  |  |
| DD-Peptidase family |  |
| YP_192994.1 | DDPept |
| YP_193653.1 | DDPept |
| YP_193750.1 | DDPept |
| YP_193887.1 | DDPept |
| YP_194451.1 | DDPept |
| YP_194750.1 | DDPept |
|  |  |
| Clp protease family |  |
| YP_193600.1 | Clp |
|  |  |
| Lon protease family |  |
| YP_193729.1 | Lon_C |
|  |  |
| Lactobacillus_johnsonii_NCC_533 |  |
| Trypsin family |  |
| NP_964086.1 | Tryp-PDZ |
|  |  |
| Subtilisin family |  |
| NP_965819.1 | Sub_N-Subt-PA-DUF1034-(FIVAR)^5^-Gram-pos-anch |
|  |  |
| DD-Peptidase family |  |
| NP_964782.1 | DDPept |
| NP_965496.1 | DDPept |
| NP_965759.1 (**SPH; S93-; K96-**) | DDPept |
|  |  |
| Clp protease family |  |
| NP_964724.1 | Clp |
|  |  |
| Lon protease family |  |
| NP_964856.1 | Lon_C |
|  |  |
| Lactobacillus_sakei_23K |  |
| Trypsin family |  |
| YP_394693.1 | Tryp |
|  |  |
| DD-Peptidase family |  |
| YP_394712.1 | DDPept |
| YP_395263.1 | DDPept |
| YP_395756.1 | DDPept |
|  |  |
| Clp protease family |  |
| YP_395143.1 | Clp |
|  |  |
| Lactococcus_lactis |  |
| Trypsin family |  |
| NP_268293.1 | Tryp-PDZ |
|  |  |
| DD-Peptidase family |  |
| NP_268202.1 | DDPept |
|  |  |
| Clp protease family |  |
| NP_266829.1 | Clp |
| NP_267552.1 (**SPH; D185E**) | Clp |
|  |  |
| Lon protease family |  |
| NP_268318.1 | Lon_C |
|  |  |
| Mesorhizobium_loti |  |
| Trypsin family |  |
| NP_102146.1 | Tryp-PDZ-PDZ |
| NP_103037.1 | Tryp-PDZ-PDZ |
| NP_103768.1 | Tryp-PDZ |
| NP_104443.1 | Tryp |
| NP_104444.1 | Tryp |
| NP_105757.1 | Tryp-PDZ-PDZ |
| NP_107298.1 | Tryp |
| NP_107958.1 | Tryp-PDZ-PDZ |
|  |  |
| Subtilisin family |  |
| NP_104000.1 | Subt |
| NP_105834.1 | Subt |
|  |  |
| DD-Peptidase family |  |
| NP_103134.1 | DDPept |
| NP_103453.1 | DDPept |
| NP_104118.1 | DDPept |
| NP_104119.1 | DDPept |
| NP_107127.1 | DDPept |
| NP_107250.1 | DDPept |
| NP_107604.1 | DDPept |
|  |  |
| Clp protease family |  |
| NP_102489.1 | Clp |
| NP_108564.1 | Clp |
| NP_108601.1 (**SPH; D185E**) | Clp |
|  |  |
| Lon protease family |  |
| NP_108566.1 | LON-AAA-Lon_C |
|  |  |
| Methanococcoides_burtonii_DSM_6 |  |
| Subtilisin family |  |
| YP_565583.1 | Subt-(fn3)^3^-(PKD)^3^ |
| YP_565629.1 | Subt |
|  |  |
| Lon protease family |  |
| YP_565579.1 (Lon_B) | Mg_chelatase-Lon_C |
|  |  |
| Methanococcus_jannaschii |  |
| Lon protease family |  |
| NP_248318.1 | Lon_C |
| NP_248420.1 (Lon_B) | Mg_chelatase-Lon_C |
|  |  |
| Methanosarcina_acetivorans |  |
| Subtilisin family |  |
| NP_616940.1 | Subt-NosD |
| NP_617222.1 (**SPH; S325-**) | Subt-(TPR_1-TPR_2)^6^ |
| NP_619099.1 | Subt |
|  |  |
| DD-Peptidase family |  |
| NP_616281.1 | DDPept |
|  |  |
| Lon protease family |  |
| NP_616787.1 | LON-AAA-Lon_C |
| NP_618179.1 (Lon_B) | Mg_chelatase-Lon_C |
|  |  |
| Methanosarcina_mazei |  |
| Subtilisin family |  |
| NP_633000.1 | Subt |
| NP_635087.1 | Subt |
|  |  |
| DD-Peptidase family |  |
| NP_635181.1 | DDPept |
|  |  |
| Lon protease family |  |
| NP_632152.1 (Lon_B) | Mg_chelatase-Lon_C |
| NP_635142.1 | LON-AAA-Lon_C |
|  |  |
| Mycobacterium_leprae |  |
| Trypsin family |  |
| NP_301252.1 | Tryp |
| NP_301791.1 | Tryp-PDZ |
| NP_302493.1 | Colicin_V-Tryp |
| NP_302696.1 | Tryp-PDZ |
|  |  |
| Subtilisin family |  |
| NP_301157.1 | Subt |
| NP_302071.1 | Subt |
| NP_302623.1 | Subt |
|  |  |
| DD-Peptidase family |  |
| NP_301215.1 | DDPept |
| NP_302211.1 | DDPept |
|  |  |
| Clp protease family |  |
| NP_302040.1 | Clp |
| NP_302041.1 | Clp |
|  |  |
| Lon protease family |  |
| NP_301531.1 | Lon_C |
|  |  |
| Mycobacterium_tuberculosis_CDC1551 |  |
| Trypsin family |  |
| NP_334543.1 | Tryp-PDZ |
| NP_335445.1 | Tryp-PDZ |
| NP_335510.1 | Tryp |
| NP_335704.1 | Tryp-PDZ |
| NP_338322.1 | Tryp |
| NP_338325.1 | Colicin_V-Tryp |
|  |  |
| Subtilisin family |  |
| NP_334714.1 | Subt |
| NP_336302.1 | Subt |
| NP_338081.1 | Subt |
| NP_338552.1 | Subt |
|  |  |
| DD-Peptidase family |  |
| NP_334819.1 (**SPH; S93D; K96I**) | DDPept |
| NP_335363.1 | DDPept |
| NP_335863.1 | DDPept |
| NP_335997.1 | DDPept |
| NP_336222.1 | DDPept |
| NP_336229.1 | DDPept |
| NP_336431.1 | DDPept |
| NP_336432.1 | DDPept |
| NP_336594.1 | DDPept |
| NP_336786.1 | DDPept |
| NP_337024.1 | DDPept |
| NP_337485.1 | DDPept |
| NP_338435.1 | DDPept |
|  |  |
| Clp protease family |  |
| NP_337021.1 | Clp |
| NP_337022.1 | Clp |
|  |  |
| Lon protease family |  |
| NP_337813.1 | Lon_C |
|  |  |
| Nanoarchaeum_equitans |  |
| Lon protease family |  |
| NP_963637.1 (Lon_B) | Sigma54_activat-Lon_C |
|  |  |
| Natronomonas_pharaonis |  |
| Trypsin family |  |
| YP_325990.1 | Tryp |
|  |  |
| Subtilisin family |  |
| YP_326498.1 | Subt-PKD-PKD |
| YP_326977.1 | Subt |
| YP_331041.1 | Subt |
|  |  |
| Lon protease family |  |
| YP_326115.1 (Lon_B) | Sigma54_activat-Lon_C |
|  |  |
| Neisseria_meningitidis_MC58 |  |
| Trypsin family |  |
| NP_273577.1 | Tryp-PDZ-PDZ |
|  |  |
| Subtilisin family |  |
| NP_274963.1 | Subt-Autotrans |
|  |  |
| Clp protease family |  |
| NP_274331.1 | Clp |
|  |  |
| Lon protease family |  |
| NP_274255.1 | LON-AAA-Lon_C |
|  |  |
| Novosphingobium_aromaticivorans_DSM_12444 |  |
| Trypsin family |  |
| YP_497668.1 | Tryp |
| YP_498600.1 | Tryp-PDZ-PDZ |
|  |  |
| Subtilisin family |  |
| YP_496867.1 | Subt |
| YP_497111.1 | Subt |
|  |  |
| DD-Peptidase family |  |
| YP_495328.1 | DDPept |
| YP_495497.1 | DDPept |
| YP_495562.1 | DDPept |
| YP_496149.1 | DDPept |
| YP_496240.1 | DDPept |
| YP_497067.1 | DDPept |
| YP_497298.1 | DDPept |
|  |  |
| Clp protease family |  |
| YP_498324.1 | Clp |
|  |  |
| Lon protease family |  |
| YP_496639.1 | LON-AAA-Lon_C |
|  |  |
| Oceanobacillus_iheyensis |  |
| Trypsin family |  |
| NP_691504.1 | Tryp-PDZ |
| NP_692021.1 | Tryp |
| NP_694369.1 | Tryp-PDZ |
|  |  |
| Subtilisin family |  |
| NP_691157.1 | Sub_N-Subt-PA-(Dockerin_1)_2_ |
| NP_691654.1 | Sub_N-Subt-PA |
| NP_693252.1 | Sub_N-Subt-Cleaved_Adhesin |
| NP_693296.1 | Subt |
| NP_693519.1 | Sub_N-Subt |
| NP_693771.1 | Sub_N-Subt |
| NP_693854.1 | Subt-PA-DUF1034 |
| NP_693905.1 | Subt-PA |
|  |  |
| DD-Peptidase family |  |
| NP_691206.1 | DDPept |
| NP_691588.1 | DDPept |
| NP_693715.1 | DDPept |
| NP_694282.1 | DDPept |
|  |  |
| Clp protease family |  |
| NP_692533.1 | Clp |
| NP_693377.1 | Clp |
|  |  |
| Lon protease family |  |
| NP_692997.1 | LON-AAA-Lon_C |
|  |  |
| Pelodictyon_luteolum_DSM_273 |  |
| Trypsin family |  |
| YP_374315.1 | Tryp |
| YP_374752.1 | Tryp-(Sel1)^6^ |
| YP_375369.1 | Tryp-PDZ-PDZ |
|  |  |
| Subtilisin family |  |
| YP_374295.1 | Subt |
|  |  |
| DD-Peptidase family |  |
| YP_374370.1 | DDPept |
|  |  |
| Clp protease family |  |
| YP_374316.1 | Clp |
|  |  |
| Lon protease family |  |
| YP_374229.1 (Lon_B) | Lon_C |
|  |  |
| Photorhabdus_luminescens |  |
| Trypsin family |  |
| NP_930234.1 | Tryp |
| NP_931218.1 | Tryp-PDZ-PDZ |
| NP_931219.1 | Tryp-PDZ |
|  |  |
| Subtilisin family |  |
| NP_927988.1 | (HemolysinCabind)_3_-Subt-P_proprotein-HemolysinCabind |
| NP_930054.1 | Subt |
| NP_931624.1 | Subt |
|  |  |
| DD-Peptidase family |  |
| NP_927528.1 | DDPept |
| NP_927530.1 | DDPept |
| NP_927531.1 | DDPept |
| NP_927532.1 | DDPept |
| NP_927533.1 | DDPept |
| NP_928174.1 | DDPept |
| NP_931644.1 | DDPept |
|  |  |
| Clp protease family |  |
| NP_931074.1 | Clp |
|  |  |
| Lon protease family |  |
| NP_931072.1 | LON-AAA-Lon_C |
|  |  |
| Propionibacterium_acnes_KPA17120 |  |
| Trypsin family |  |
| YP_056781.1 | Colicin_V-Tryp |
| YP_056971.1 | Tryp-PDZ |
|  |  |
| Subtilisin family |  |
| YP_055311.1 | Subt |
|  |  |
| DD-Peptidase family |  |
| YP_055843.1 | DDPept |
| YP_056216.1 | DDPept |
|  |  |
| Clp protease family |  |
| YP_056273.1 | Clp |
| YP_056274.1 | Clp |
|  |  |
| Lon protease family |  |
| YP_056017.1 | Lon_C |
|  |  |
| Pseudoalteromonas_haloplanktis_TAC125 |  |
| Trypsin family |  |
| YP_341026.1 | Tryp-PDZ-PDZ |
| YP_341027.1 | Tryp |
|  |  |
| Subtilisin family |  |
| YP_338620.1 | Subt-PA |
| YP_341139.1 | Subt-PPC-PPC |
|  |  |
| DD-Peptidase family |  |
| YP_341139.1 | DDPept |
| YP_339054.1 | DDPept |
| YP_339389.1 | DDPept |
|  |  |
| Clp protease family |  |
| YP_340561.1 | Clp |
|  |  |
| Lon protease family |  |
| YP_339554.1 (Lon_B) | Lon_C |
| YP_340559.1 | LON-AAA-Lon_C |
|  |  |
| Pseudomonas_aeruginosa |  |
| Trypsin family |  |
| NP_249457.1 | Tryp-PDZ-PDZ |
| NP_253136.1 | Tryp-PDZ |
|  |  |
| Subtilisin family |  |
| NP_249933.1 | Subt |
| NP_252225.1 | Subt-Autotrans |
|  |  |
| DD-Peptidase family |  |
| NP_249738.1 | DDPept |
| NP_250488.1 (**SPH; S93D**) | DDPept |
| NP_250918.1 | DDPept |
| NP_251005.1 | DDPept |
| NP_252799.1 | DDPept |
| NP_253037.1 | DDPept |
| NP_254229.1 | DDPept |
|  |  |
| Clp protease family |  |
| NP_250492.1 | Clp |
| NP_250879.1 (**SPH; D185Y**) | Clp |
| NP_252016.1 | Clp |
|  |  |
| Lon protease family |  |
| NP_249470.1 | LON-AAA-Lon_C |
| NP_250494.1 | LON-AAA-Lon_C |
| NP_253266.1 (Lon_B) | Lon_C |
|  |  |
| Pseudomonas_fluorescens_Pf-5 |  |
| Trypsin family |  |
| YP_258064.1 | Tryp-PDZ |
| YP_258579.1 | Tryp-PDZ-PDZ |
|  |  |
| Subtilisin family |  |
| YP_260308.1 | Subt-Autotrans |
| YP_260309.1 | Subt-Autotrans |
|  |  |
| DD-Peptidase family |  |
| YP_258410.1 | DDPept |
| YP_258471.1 | DDPept |
| YP_259747.1 (**SPH; S93A; K96Q**) | DDPept |
| YP_259881.1 | DDPept |
| YP_260730.1 | DDPept |
| YP_261070.1 (**SPH; S93C**) | DDPept |
| YP_261151.1 | DDPept |
|  |  |
| Clp protease family |  |
| YP_259115.1 (**SPH; D185E**) | Clp |
| YP_261087.1 | Clp |
| YP_261821.1 (**SPH; D185E**) | Clp |
|  |  |
| Lon protease family |  |
| YP_261085.1 | LON-AAA-Lon_C |
| YP_262003.1 | LON-AAA-Lon_C |
| YP_262406.1 (Lon_B) | Lon_C |
| YP_262424.1 | Lon_C |
|  |  |
| Pseudomonas_putida_KT2440 |  |
| Trypsin family |  |
| NP_743461.1 | Tryp-PDZ |
| NP_743588.1 | Tryp-PDZ-PDZ |
|  |  |
| Subtilisin family |  |
| NP_746232.1 | Subt |
| NP_747027.1 | (HemolysinCabind)_2_-Subt-P_proprotein |
|  |  |
| DD-Peptidase family |  |
| NP_743050.1 | DDPept |
| NP_743288.1 | DDPept |
| NP_744106.1 | DDPept |
| NP_745020.1 | DDPept |
|  |  |
| Clp protease family |  |
| NP_744449.1 | Clp |
| NP_745189.1 (**SPH; D185E**) | Clp |
| NP_745410.1 (**SPH; D185Y**) | Clp |
|  |  |
| Lon protease family |  |
| NP_742841.1 (Lon_B) | Lon_C |
| NP_743601.1 | LON-AAA-Lon_C |
| NP_744451.1 | LON-AAA-Lon_C |
| NP_746753.1 | Lon_C |
|  |  |
| Pseudomonas_syringae_phaseolicola_1448A |  |
| Trypsin family |  |
| YP_273108.1 | TerD-Tryp |
| YP_276088.1 | Tryp-PDZ-PDZ |
| YP_276257.1 | Tryp-PDZ |
|  |  |
| Subtilisin family |  |
| YP_272486.1 | Subt |
| YP_273777.1 | Subt |
|  |  |
| DD-Peptidase family |  |
| YP_273663.1 | DDPept |
| YP_274619.1 | DDPept |
| YP_275443.1 | DDPept |
| YP_276178.1 | DDPept |
|  |  |
| Clp protease family |  |
| YP_273935.1 | Clp |
|  |  |
| Lon protease family |  |
| YP_273937.1 | LON-AAA-Lon_C |
| YP_276077.1 | LON-AAA-Lon_C |
| YP_276406.1 (Lon_B) | Lon_C |
| YP_276449.1 | Lon_C |
|  |  |
| Pyrobaculum_aerophilum |  |
| Trypsin family |  |
| NP_560260.1 | Tryp-PDZ |
|  |  |
| Subtilisin family |  |
| NP_558788.1 | Subt |
| NP_559678.1 (**SPH; D137-; H168-**) | Subt |
|  |  |
| DD-Peptidase family |  |
| NP_560593.1 | DDPept |
|  |  |
| Lon protease family |  |
| NP_560787.1 | Lon_C |
|  |  |
| Pyrococcus_abyssi |  |
| DD-Peptidase family |  |
| NP_125831.1 | DDPept |
|  |  |
| Clp protease family |  |
| NP_126341.1 (**SPH; H136A**) | Clp-NfeD |
|  |  |
| Lon protease family |  |
| NP_126400.1 | Lon_C |
| NP_127256.1 (Lon_B) | Sigma54_activat-Lon_C |
|  |  |
| Pyrococcus_furiosus |  |
| Subtilisin family |  |
| NP_578016.1 | Subt-PPC |
| NP_578417.1 (**SPH; D137-; H168-**) | Subt |
| NP_579399.1 | Subt-PPC-PPC |
|  |  |
| Clp protease family |  |
| NP_579262.1 | Clp-NfeD |
|  |  |
| Lon protease family |  |
| NP_578196.1 (Lon_B) | Mg_chelatase-Sigma54_activat-Lon_C |
| NP_579167.1 | Lon_C |
|  |  |
| Ralstonia_eutropha_JMP134 |  |
| Subtilisin family |  |
| YP_293577.1 | Subt |
|  |  |
| DD-Peptidase family |  |
| YP_293233.1 | DDPept |
|  |  |
| Ralstonia_solanacearum |  |
| Trypsin family |  |
| NP_519179.1 | Tryp-PDZ-PDZ |
| NP_521053.1 | Tryp-PDZ |
| NP_521261.1 | Tryp |
|  |  |
| Subtilisin family |  |
| NP_520774.1 | Subt |
| NP_520775.1 | Subt |
| NP_521222.1 | Subt |
|  |  |
| DD-Peptidase family |  |
| NP_519886.1 (**SPH;K96Q**) | DDPept |
|  |  |
| Clp protease family |  |
| NP_519832.1 | Clp |
|  |  |
| Lon protease family |  |
| NP_519834.1 | LON-AAA-Lon_C |
|  |  |
| Rhodopseudomonas_palustris_CGA009 |  |
| Trypsin family |  |
| NP_946693.1 | Tryp |
| NP_947240.1 | Tryp |
| NP_947274.1 | Tryp-PDZ-PDZ |
| NP_947769.1 | Tryp |
| NP_948560.1 | Tryp-PDZ-PDZ |
| NP_948653.1 | Tryp-PDZ |
| NP_948826.1 | Tryp-PDZ-PDZ |
| NP_949906.1 | Tryp-PDZ-PDZ |
|  |  |
| Subtilisin family |  |
| NP_947281.1 | Subt-Autotrans |
| NP_949347.1 | Subt-Autotrans |
|  |  |
| DD-Peptidase family |  |
| NP_945715.1 | DDPept |
| NP_945813.1 | DDPept |
| NP_946954.1 | DDPept |
| NP_947223.1 | DDPept |
| NP_947493.1 | DDPept |
| NP_948152.1 | DDPept |
| NP_948623.1 | DDPept |
|  |  |
| Clp protease family |  |
| NP_948302.1 | Clp |
|  |  |
| Lon protease family |  |
| NP_948300.1 | LON-AAA-Lon_C |
|  |  |
| Rubrobacter_xylanophilus_DSM_9941 |  |
| Trypsin family |  |
| YP_643190.1 | Tryp |
| YP_643229.1 | Colicin_V-Tryp |
| YP_643384.1 | Tryp-PDZ |
| YP_643449.1 | Tryp-PDZ |
| YP_643512.1 | Tryp |
| YP_643608.1 | Tryp |
| YP_645742.1 | Tryp-PDZ |
|  |  |
| Subtilisin family |  |
| YP_644071.1 | Subt |
| YP_644944.1 | Subt |
| YP_645652.1 | Subt |
|  |  |
| DD-Peptidase family |  |
| YP_643409.1 | DDPept |
| YP_644484.1 | DDPept |
| YP_644860.1 | DDPept |
|  |  |
| Clp protease family |  |
| YP_644311.1 | Clp |
|  |  |
| Lon protease family |  |
| YP_644688.1 (Lon_B) | Lon_C |
|  |  |
| Salinibacter_ruber_DSM_13855 |  |
| Trypsin family |  |
| YP_445732.1 | Tryp-PDZ |
| YP_446170.1 | Tryp-PDZ |
|  |  |
| Subtilisin family |  |
| YP_444708.1 | Subt |
| YP_445143.1 | Subt |
| YP_445525.1 | Subt |
| YP_446305.1 | Subt |
| YP_446403.1 | Subt-fn3 |
|  |  |
| DD-Peptidase family |  |
| YP_444518.1 | Glyco_hydro_3-Glyco_hydro_3_C-DDPept |
| YP_445028.1 | DDPept |
| YP_445083.1 | DDPept |
| YP_446813.1 | DDPept |
|  |  |
| Clp protease family |  |
| YP_445563.1 (**SPH; D185N**) | Clp |
| YP_446772.1 (**SPH; D185N**) | Clp |
|  |  |
| Shewanella_oneidensis |  |
| Trypsin family |  |
| NP_719473.1 | Tryp-PDZ-PDZ |
| NP_719474.1 | Tryp-PDZ |
|  |  |
| Subtilisin family |  |
| NP_715829.1 | Subt |
| NP_716498.1 | Subt-P_proproteinein-PKD |
| NP_717522.1 | Sub_N-Subt-PA-PPC |
| NP_718668.1 | Sub_N-Subt-PA-PPC-P_proproteinein |
| NP_718856.1 | Subt-PA |
| NP_719336.1 | Subt-PA |
| NP_720056.1 | Subt-PA |
|  |  |
| DD-Peptidase family |  |
| NP_716543.1 | DDPept |
| NP_717978.1 | DDPept |
|  |  |
| Clp protease family |  |
| NP_717403.1 | Clp |
| NP_718533.1 (**SPH; D185E**) | Clp |
|  |  |
| Lon protease family |  |
| NP_717405.1 | LON-AAA-Lon_C |
| NP_718945.1 (Lon_B) | Lon_C |
|  |  |
| Sinorhizobium_meliloti |  |
| Trypsin family |  |
| NP_435856.1 | Tryp-PDZ |
|  |  |
| Subtilisin family |  |
| NP_435320.1 | GRP-Subt |
| NP_436293.1 | Subt |
|  |  |
| DD-Peptidase family |  |
| NP_436314.1 | DDPept |
| NP_436315.1 | DDPept |
|  |  |
| Staphylococcus_aureus_COL |  |
| Trypsin family |  |
| YP_185894.1 | Tryp |
| YP_185922.1 | Tryp |
| YP_186611.1 | Tryp |
| YP_186692.1 | Tryp |
| YP_186693.1 | Tryp |
| YP_186694.1 | Tryp |
| YP_186695.1 | Tryp |
| YP_186696.1 | Tryp |
| YP_186697.1 | Tryp |
|  |  |
| Subtilisin family |  |
| YP_186702.1 | Subt |
|  |  |
| DD-Peptidase family |  |
| YP_185930.1 | DDPept |
| YP_187245.1 | DDPept |
|  |  |
| Clp protease family |  |
| YP_185261.1 (**SPH; D185E**) | Clp |
| YP_185707.1 | Clp |
|  |  |
| Staphylococcus_epidermidis_ATCC_12228 |  |
| Trypsin family |  |
| NP_764278.1 **(SPH; H57-)** | Tryp |
| NP_764960.1 | Tryp |
| NP_765098.1 | Tryp |
|  |  |
| DD-Peptidase family |  |
| NP_764309.1 | DDPept |
| NP_765163.1 | DDPept |
| NP_765562.1 | DDPept |
|  |  |
| Clp protease family |  |
| NP_764106.1 | Clp |
|  |  |
| Streptococcus_agalactiae_2603 |  |
| Trypsin family |  |
| NP_689159.1 | Tryp |
|  |  |
| Subtilisin family |  |
| NP_687450.1 | Subt-PA-DUF1034 |
| NP_687694.1 | Subt-DUF1034 |
| NP_689039.1 | YSIRK_sig-Subt-PA-DUF1034-(FIR)^3^ |
|  |  |
| DD-Peptidase family |  |
| NP_687676.1 | DDPept |
| NP_688719.1 | DDPept |
|  |  |
| Clp protease family |  |
| NP_688576.1 | Clp |
|  |  |
| Streptococcus_mutans |  |
| Trypsin family |  |
| NP_722446.1 | Tryp-PDZ |
|  |  |
| DD-Peptidase family |  |
| NP_720848.1 | DDPept |
| NP_721297.1 | DDPept |
|  |  |
| Clp protease family |  |
| NP_722005.1 | Clp |
|  |  |
| Streptococcus_pneumoniae_TIGR4 |  |
| Trypsin family |  |
| NP_344916.1 | Tryp-CW_binding-CW_binding |
| NP_346646.1 | Tryp |
|  |  |
| Subtilisin family |  |
| NP_345151.1 | Sub_N-Subt-PA-DUF1034-Gram_pos_anchor |
|  |  |
| DD-Peptidase family |  |
| NP_345902.1 | DDPept |
|  |  |
| Clp protease family |  |
| NP_345245.1 | Clp |
|  |  |
| Streptomyces_avermitilis |  |
| Trypsin family |  |
| NP_822175.1 | Pro_Al_prot-Tryp-CBM_5_12 |
| NP_823210.1 | Tryp |
| NP_823619.1 | Tryp |
| NP_824291.1 | Tryp-PDZ |
| NP_825221.1 | Tryp-(FG-GAP)_4_ |
| NP_825222.1 | Tryp |
| NP_825262.1 | Tryp |
| NP_825400.1 | Tryp |
| NP_825772.1 | Colicin_V-Tryp |
| NP_827192.1 | Colicin_V-Tryp |
| NP_827384.1 | Tryp |
| NP_827728.1 | Pro_Al_prot-Tryp |
| NP_827729.1 | (Pro_Al_prot)_2_-Tryp |
| NP_828673.1 | Pro_Al_prot-Tryp |
|  |  |
| Subtilisin family |  |
| NP_821719.1 | Subt |
| NP_823710.1 | Subt |
| NP_823716.1 | Subt |
| NP_823977.1 | Sub_N-Subt |
| NP_824495.1 | Subt-BNR-BNR |
| NP_825752.1 | Subt |
| NP_826577.1 | Subt-PPC-PPC |
| NP_826886.1 | Subt |
| NP_826898.1 | Subt-PA |
| NP_827126.1 (**SPH; H168Y**) | Subt |
| NP_827627.1 | Subt |
| NP_827628.1 | Subt |
| NP_827752.1 | Sub_N-Subt |
| NP_827825.1 | Subt |
| NP_827909.1 | Subt |
|  |  |
| DD-Peptidase family |  |
| NP_822436.1 | DDPept |
| NP_822479.1 | DDPept |
| NP_822959.1 | DDPept |
| NP_823839.1 | DDPept |
| NP_824788.1 | DDPept |
| NP_824819.1 (**SPH; Y190K**) | (Cond-AMPbind-PPbind)^3^-DDPept |
| NP_825460.1 | DDPept |
| NP_825629.1 | DDPept |
| NP_825632.1 | DDPept |
| NP_826181.1 | DDPept |
| NP_826941.1 | DDPept |
| NP_826968.1 | DDPept |
| NP_827095.1 | DDPept |
|  |  |
| Clp protease family |  |
| NP_822321.1 | Clp |
| NP_822322.1 | Clp |
| NP_826624.1 | Clp |
| NP_826625.1 | Clp |
| NP_828277.1 | Clp |
|  |  |
| Lon protease family |  |
| NP_824142.1 | LON-AAA-Lon_C |
| NP_826319.1 | Lon_C |
|  |  |
| Sulfolobus_acidocaldarius_DSM_639 |  |
| Trypsin family |  |
| NP_343943.1 | Tryp-PDZ |
| YP_255674.1 | Tryp-PDZ |
|  |  |
| Subtilisin family |  |
| NP_343565.1 | Subt |
| NP_343890.1 | Subt |
| YP_255789.1 | Subt |
| YP_256856.1 | Subt |
| YP_256945.1 (**SPH; D137V; H168Q**) | Subt |
|  |  |
| Sulfolobus_solfataricus |  |
| Trypsin family |  |
| NP_343943.1 | Tryp-PDZ |
|  |  |
| Subtilisin family |  |
| NP_343565.1 | Subt |
| NP_343890.1 | Subt |
|  |  |
| Sulfolobus_tokodaii |  |
| Trypsin family |  |
| NP_376823.1 | Tryp-PDZ |
|  |  |
| Subtilisin family |  |
| NP_378163.1 | Subt |
| NP_378365.1 | Subt |
| NP_378492.1 | Subt |
|  |  |
| Symbiobacterium_thermophilum_IAM14863 |  |
| Trypsin family |  |
| YP_073997.1 | Tryp |
| YP_074048.1 (**SPH; H57Q; D102F; S195A**) | Tryp |
| YP_074246.1 | Tryp |
| YP_075854.1 | Tryp |
|  |  |
| Subtilisin family |  |
| YP_073891.1 | Subt |
| YP_074316.1 | Sub_N-Subt |
| YP_074547.1 | Subt- Cleaved_Adhesin - fn3-PKD-PKD |
| YP_074584.1 | Sub_N-Subt-PA |
| YP_074862.1 | Subt |
|  |  |
| DD-Peptidase family |  |
| YP_074131.1 | DDPept |
| YP_074464.1 | DDPept |
| YP_074550.1 | DDPept |
| YP_074764.1 | DDPept |
| YP_074809.1 | DDPept |
| YP_075397.1 | DDPept |
| YP_075850.1 | DDPept |
| YP_076204.1 | DDPept |
|  |  |
| Clp protease family |  |
| YP_074187.1 | Clp |
| YP_074743.1 | Clp |
| YP_075387.1 | Clp |
|  |  |
| Lon protease family |  |
| YP_074189.1 | Sigma54_activat-AAA-Lon_C |
| YP_074190.1 | LON-AAA-Lon_C |
| YP_077141.1 | Sigma54_activat-AAA-Lon_C |
|  |  |
| Synechococcus_CC9605 |  |
| Trypsin family |  |
| YP_381285.1 | Tryp-PDZ |
| YP_382367.1 | Tryp-PDZ |
| YP_382607.1 | Tryp-PDZ |
|  |  |
| Subtilisin family |  |
| YP_382882.1 | (W_rich_C)_2_-PPC-PPC-Subt |
|  |  |
| DD-Peptidase family |  |
| YP_381800.1 | DDPept |
| YP_382201.1 | DDPept |
| YP_382246.1 | DDPept |
|  |  |
| Clp protease family |  |
| YP_380399.1 | Clp |
| YP_381163.1 | Clp |
| YP_381164.1 (**SPH; S111G; D185M**) | Clp |
| YP_381749.1 | Clp |
|  |  |
| Synechocystis_PCC6803 |  |
| Trypsin family |  |
| NP_440115.1 | Tryp-PDZ |
| NP_440705.1 | Tryp-PDZ |
| NP_441326.1 | Tryp-PDZ |
|  |  |
| Subtilisin family |  |
| NP_442752.1 | Subt |
|  |  |
| DD-Peptidase family |  |
| NP_441611.1 | DDPept |
| NP_441897.1 | DDPept |
| NP_442589.1 | DDPept |
|  |  |
| Clp protease family |  |
| NP_441889.1 (**SPH; S111G; H136N; D185T**) | Clp |
| NP_441890.1 | Clp |
| NP_442765.1 | Clp |
| NP_442796.1 | Clp |
|  |  |
| Thermoanaerobacter_tengcongensis |  |
| Trypsin family |  |
| NP_624088.1 | Tryp-PDZ |
| NP_624198.1 | Tryp-PDZ |
|  |  |
| Subtilisin family |  |
| NP_622243.1 | Subt |
| NP_622477.1 | Sub_N-Subt-PA |
| NP_624131.1 | Subt-PA-DUF1034-(Big2)^3^-(SLH)^3^ |
|  |  |
| DD-Peptidase family |  |
| NP_621910.1 | DDPept |
| NP_621928.1 | DDPept |
|  |  |
| Clp protease family |  |
| NP_622290.1 | Clp |
| NP_622999.1 | Clp |
|  |  |
| Lon protease family |  |
| NP_622292.1 | LON-AAA-Lon_C |
| NP_623361.1 (Lon_B) | Lon_C |
|  |  |
| Thermococcus_kodakaraensis_KOD1 |  |
| Subtilisin family |  |
| YP_182489.1 | Subt |
| YP_184088.1 | Subt |
| YP_184102.1 | Subt-PPC-PPC |
|  |  |
| Lon protease family |  |
| YP_183677.1 (Lon_B) | Sigma54_activat-AAA-Lon_C |
| YP_184581.1 | Lon_C |
|  |  |
| Thermoplasma_acidophilum |  |
| Subtilisin family |  |
| NP_394205.1 | Thermopsin-Subt |
| NP_394436.1 | Subt |
|  |  |
| Lon protease family |  |
| NP_394540.1 (Lon_B) | Sigma54_activat-AAA-Lon_C |
|  |  |
| Thermus_thermophilus_HB27 |  |
| Trypsin family |  |
| YP_004392.1 | Tryp-PDZ |
| YP_004925.1 | Tryp |
| YP_005874.1 | Tryp |
|  |  |
| Subtilisin family |  |
| YP_004347.1 | Subt |
|  |  |
| DD-Peptidase family |  |
| YP_004371.1 | DDPept |
| YP_004379.1 | DDPept |
|  |  |
| Clp protease family |  |
| YP_004225.1 | Clp |
|  |  |
| Lon protease family |  |
| YP_004393.1 | LON-AAA-Lon_C |
| YP_004719.1 | LON-AAA-Lon_C |
| YP_005944.1 (Lon_B) | Lon_C |
|  |  |
| Thiomicrospira_crunogena_XCL-2 |  |
| Trypsin family |  |
| YP_391001.1 | Tryp-PDZ-PDZ |
|  |  |
| Subtilisin family |  |
| YP_391874.1 | Subt |
|  |  |
| DD-Peptidase family |  |
| YP_390608.1 | DDPept |
| YP_391341.1 | DDPept |
|  |  |
| Clp protease family |  |
| YP_391446.1 | Clp |
|  |  |
| Lon protease family |  |
| YP_391224.1 (Lon_B) | Lon_C |
| YP_391448.1 | LON-AAA-Lon_C |
| YP_391814.1 | LON-AAA-Lon_C |
|  |  |
| Treponema_denticola_ATCC_35405 |  |
| Trypsin family |  |
| NP_971363.1 | Tryp |
| NP_971949.1 | Tryp |
| NP_972332.1 | Tryp |
| NP_972569.1 | Tryp |
| NP_972900.1 | Tryp-PDZ-PDZ |
|  |  |
| DD-Peptidase family |  |
| NP_972696.1 | DDPept |
|  |  |
| Clp protease family |  |
| NP_972277.1 | Clp |
| NP_972987.1 | Clp |
|  |  |
| Lon protease family |  |
| NP_971283.1 | LON-AAA-Lon_C |
|  |  |
| Xanthomonas_campestris |  |
| Trypsin family |  |
| NP_636643.1 | Tryp-PDZ-PDZ |
| NP_638169.2 | Tryp |
| NP_639238.1 | Tryp-PDZ |
|  |  |
| Subtilisin family |  |
| NP_636051.1 | Subt-PPC |
| NP_636242.1 | Subt-PPC |
| NP_636243.1 | Subt |
| NP_636245.2 | Subt-PPC |
| NP_636672.2 | Subt-Autotrans |
| NP_636837.2 | Subt |
| NP_637033.2 | Subt |
| NP_637390.1 | Subt-Autotrans |
| NP_638014.1 | Subt |
| NP_638022.1 | Subt-P_proprotein |
|  |  |
| DD-Peptidase family |  |
| NP_637984.1 | DDPept |
| NP_638221.1 | DDPept |
| NP_638386.1 | DDPept |
|  |  |
| Clp protease family |  |
| NP_636356.1 | Clp |
|  |  |
| Lon protease family |  |
| NP_636358.1 | LON-AAA-Lon_C |
|  |  |
| Xylella_fastidiosa |  |
| Trypsin family |  |
| NP_297578.1 | Tryp-PDZ |
| NP_299520.1 | Tryp-PDZ-PDZ |
|  |  |
| Subtilisin family |  |
| NP_297560.1 | Subt-Autotrans |
| NP_298316.2 | Subt-Autotrans |
| NP_299137.1 | Subt-Autotrans |
|  |  |
| DD-Peptidase family |  |
| NP_298910.1 | DDPept |
|  |  |
| Clp protease family |  |
| NP_297801.1 | Clp |
| NP_298477.1 | Clp |
|  |  |
| Lon protease family |  |
| NP_298479.1 | LON-AAA-Lon_C |

# Co-exisiting domains: **Tryp**-Trypsin; **Subt**-Subtilisin; **DDPept**- DD-peptidase; **Clp**- Clp protease; **Lon**_**C**- Lon protease; **AAA**- ATPase family associated with various cellular activities (PF00004); **ABC_tran**- ABC transporter (PF00005); **AMP-binding**- AMP-binding enzyme (PF00501); **ANF_receptor**- Receptor family ligand binding region (PF01094); Autotransporter- Autotransporter beta-domain (PF03797); **Big_2**- Bacterial Ig-like domain (group 2) (PF02368); **BNR**- BNR/Asp-box repeat (PF02012); **CARDB**- Cell adhesion related domain found in bacteria (PF07705); **Colicin_V**- Colicin V production protein (PF02674); **CBM_5_12**- Carbohydrate binding domain (PF02839); **Cleaved_Adhesin**- Cleaved Adhesin Domain (PF07675); **Cond(ensation)**- Condensation domain (PF00668); **CUB**- CUB domain (PF00431); **CW_binding_1**- Putative cell wall binding repeat (PF01473); **DnaB_C**- DnaB-like helicase C terminal domain (PF03796); Dockerin_1- Dockerin type I repeat (PF00404); **DUF1034**- Domain of unknown function (PF06280); **DUF11**- Domain of unknown function (PF01345); **DUF1343**- Protein of unknown function (PF07075); **FG-GAP**- FG-GAP repeat (PF01839); **Endonuclease_NS**- DNA/RNA non-specific endonuclease (PF01233); **FHA**- FHA (Forkhead-associated) domain (PF00498); **FIVAR**- Uncharacterised Sugar-binding Domain (PF07554); **fn3**- Fibronectin type III domain (PF00041); **Glyco_hydr_3**- Glycosyl hydrolase family 3 N terminal domain (PF00933); **Glyc_hydr_3_C**- Glycosyl hydrolase family 3 C terminal domain (PF01915); **Gram_pos_anchor**- Gram positive anchor (PF00746); **GRP**- Glycine rich protein family (PF07172); **Hemolys**- Hemolysin-type calcium-binding repeat (2 copies) (PF00353); **LON**- ATP-dependent protease La (LON) domain (PF02190); **Mg_chelat**- Magnesium chelatase, subunit ChlI (PF01078); **Nfed**- Nfed-like (PF01957); **NosD**- Periplasmic copper-binding protein (NosD) (PF05048); **P_proprotein**- Proprotein convertase P-domain (PF01483); **PA**– Protease associated domain (PF02225); **PDZ**– PDZ domain (PF00595); **PKD**- PKD domain (PF00801); **PP-binding**- Phosphopantetheine attachment site (PF00550); **PPC**- Bacterial pre-peptidase C-terminal domain (PF04151); **Pro_Al_prot**- Alpha-lytic protease prodomain (PF02983); **Sel**1- Sel1 repeat (PF08238); **SCP**- SCP-like extracellular protein (PF00188); **Sigma54_activat**- Sigma-54 interaction domain (PF00158); **SLH**- S-layer homology domain (PF00395); **Sub(tilisin)_N**- Subtilisin N-terminal region (PF005922); **TerD**- Bacterial stress protein (PF02342); **Thermopsin**- Thermopsin (PF05317); **W_rich_C**- Tryptophan-rich Synechocystis species C-terminal domain (PF07483)
