# Supplementary material for: Genome-wide survey of prokaryotic serine proteases: Analysis of distribution and domain architectures of five serine protease families in prokaryotes
Source: BMC Genomics. 2008 Nov 19;9:549. doi: 10.1186/1471-2164-9-549 (PMC2605481; doi:10.1186/1471-2164-9-549)
Supplement: Additional file 2 — A list of the various prokaryotic genomes considered for the present study. The species abbreviations employed in the illustrations of the phylogenetic trees accompany the species name in parentheses. A list of the genomes in which serine protease-like proteins were identified in the current study. The genomes have been categorized accoding to their taxonomic lineages and the abbreviated species IDs (in parenthesis) that have been suffixed to the protein identifiers in the phylogenetic trees accompany the species name. [file 1471-2164-9-549-S2.doc]

Additional file S2: A list of the various prokaryotic genomes considered for the present study. The species abbreviations employed in the illustrations of the phylogenetic trees accompany the species name in parentheses.

| **Crenarchaeota** |
| --- |
| *Aeropyrum_pernix* (Ae. pr.) |
| *Pyrobaculum_aerophilum* (Py. ar.) |
| *Sulfolobus_acidocaldarius_DSM_639* (Sf. ac.) |
| *Sulfolobus_solfataricus* (Sf. sf.) |
| *Sulfolobus_tokodaii* (Sf. tk.) |
|  |
| **Euryarchaeota** |
| *Archaeoglobus_fulgidus* (Ac. fl.) |
| *Halobacterium_sp_NRC1* (Ha. sp.) |
| *Methanococcoides_burtonii_DSM_6* (Mt. bt.) |
| *Methanococcus_jannaschii* (Mc. jn.) |
| *Methanosarcina_acetivorans* (Ms. ac.) |
| *Methanosarcina_mazei* (Ms. mz.) |
| *Natronomonas_pharaonis* (Nt. ph.) |
| *Pyrococcus_abyssi* (Pc. ab.) |
| *Pyrococcus_furiosus* (Pc. fs.) |
| *Thermococcus_kodakaraensis_KOD1* (Th. kd.) |
| *Thermoplasma_acidophilum* (Th. ac.) |
|  |
| **Nanoarchaeota** |
| *Nanoarchaeum_equitans* (Na. eq.) |
| **Bacteria** |
| **Actinobacteria** |
| *Corynebacterium_efficiens_YS-314* (Co. ef.) |
| *Corynebacterium_glutamicum_ATCC_13032_Bielefeld* (Co. gl.) |
| *Mycobacterium_leprae* (My. lp.) |
| *Mycobacterium_tuberculosis_CDC1551* (My. tb.) |
| *Propionibacterium_acnes_KPA17120* (Pp. an.) |
| *Rubrobacter_xylanophilus_DSM_9941* (Rb. xy.) |
| *Streptomyces_avermitilis* (St. av.) |
| *Symbiobacterium_thermophilum_IAM14863* (Sy. th.) |
|  |
| **Alphaproteobacteria** |
| *Agrobacterium_tumefaciens_C58_Cereon* (Ag. tm.) |
| *Bradyrhizobium_japonicum* (Br. jp.) |
| *Mesorhizobium_loti* (Ms. lt.) |
| *Novosphingobium_aromaticivorans_DSM_12444* (Nv. ar.) |
| *Rhodopseudomonas_palustris_CGA009* (Rh. pl.) |
| *Sinorhizobium_meliloti* (Si. ml.) |
|  |
| **Betaproteobacteria** |
| *Azoarcus_sp_EbN1* (Az. sp.) |
| *Bordetella_bronchiseptica* (Bo. br.) |
| *Bordetella_parapertussis* (Bo. pp.) |
| *Burkholderia_cenocepacia_AU_1054* (Bu. cc.) |
| *Burkholderia_mallei_ATCC_23344* (Bu. ml.) |
| *Burkholderia_thailandensis_E264* (Bu. th.) |
| *Chromobacterium_violaceum* (Ch. vl.) |
| *Neisseria_meningitidis_MC58* (Ne. mn.) |
| *Ralstonia_eutropha_JMP134* (Rl. eu.) |
| *Ralstonia_solanacearum* (Rl. sl.) |
|  |
| **Chlorobi** |
| *Bacteroides_thetaiotaomicron_VPI-5482* (Bc. th.) |
| *Pelodictyon_luteolum_DSM_273* (Pe. lu.) |
| *Salinibacter_ruber_DSM_13855* (Sn. ru.) |
|  |
| **Cyanobacteria** |
| *Gloeobacter_violaceus* (Gl. vl.) |
| *Synechococcus_CC9605* (Sy. cc.) |
| *Synechocystis_PCC6803* (Sn. pc.) |
|  |
| **Deinococcus-Thermus** |
| *Deinococcus_radiodurans* (Dn. rd.) |
|  |
| **Deltaproteobacteria** |
| *Bdellovibrio_bacteriovorus* (Bd. bv.) |
| *Geobacter_sulfurreducens* (Ge. sf.) |
|  |
| **Epsilonproteobacteria** |
| *Thiomicrospira_crunogena_XCL-2* (Ti. mc.) |
|  |
| **Firmicutes** |
| *Bacillus_anthracis_Ames* (Ba. an.) |
| *Bacillus_clausii_KSM-K16* (Ba. cl.) |
| *Bacillus_halodurans* (Ba. hl.) |
| *Bacillus_subtilis* (Ba. sb.) |
| *Bacillus_thuringiensis_konkukian* (Ba. tu.) |
| *Enterococcus_faecalis_V583* (En. fc.) |
| *Lactobacillus_acidophilus_NCFM* (Lb. ac.) |
| *Lactobacillus_johnsonii_NCC_533* (Lb. jh.) |
| *Lactobacillus_sakei_23K* (Lb. sk.) |
| *Lactococcus_lactis* (Lc. la.) |
| *Oceanobacillus_iheyensis* (Oc. ih..) |
| *Staphylococcus_aureus_COL* (Sp. au.) |
| *Staphylococcus_epidermidis_ATCC_12228* (Sp. ep.) |
| *Streptococcus_agalactiae_2603* (Sr. ag.) |
| *Streptococcus_mutans* (Sr. mu.) |
| *Streptococcus_pneumoniae_TIGR4* (Sr. pn.) |
| *Thermoanaerobacter_tengcongensis* (Tr. tn.) |
| *Thermus_thermophilus_HB27* (Tm. tm.) |
|  |
| **Fusobacteria** |
| *Fusobacterium_nucleatum* (Fs. nu.) |
|  |
| **Gammaproteobacteria** |
| *Escherichia_coli_O157H7_EDL933* (Es. co.) |
| *Haemophilus_influenzae* (Hm. in.) |
| *Hahella_chejuensis_KCTC_2396* (Hh. ch.) |
| *Idiomarina_loihiensis_L2TR* (Id. lh.) |
| *Photorhabdus_luminescens* (Ph. lm.) |
| *Pseudoalteromonas_haloplanktis_TAC125* (Ps. hp.) |
| *Pseudomonas_aeruginosa* (Pm. ar.) |
| *Pseudomonas_fluorescens_Pf-5* (Pm. fl.) |
| *Pseudomonas_putida_KT2440* (Pm. pt.) |
| *Pseudomonas_syringae_phaseolicola_1448A* (Pm. sy.) |
| *Shewanella_oneidensis* (Sw. on.) |
| *Xanthomonas_campestris* (Xa. ca.) |
| *Xylella_fastidiosa* (Xy. fs.) |
|  |
| **Spirochaetes** |
| *Treponema_denticola_ATCC_35405* (Tr. dn.) |
